# Characterization of genomic diversity and population structure of worldwide Duroc subpopulations and other pig breeds

**DOI:** 10.1186/s12711-025-01017-6

**Published:** 2025-12-16

**Authors:** Hui Wen, Harvey D. Blackburn, Henrique A. Mulim, Hinayah R. Oliveira, Susanne Hermesch, Ching-Yi Chen, Justin Holl, Allan P. Schinckel, Luiz F. Brito

**Affiliations:** 1 Department of Animal Sciences, Purdue University, IN 47907 West Lafayette, USA; 2 USDA ARS National Animal Germplasm Program, 1111 S. Mason St, CO 80521 Fort Collins, USA; 3 AGBU, a joint venture of NSW Department of Primary Industries and University of New England, Armidale, NSW 2351 Australia; 4 Genus PIC, Hendersonville, TN 37075 USA

## Abstract

**Background:**

Duroc is one of the most popular terminal sire pig breeds worldwide due to its greater growth rate, meat quality, feed efficiency, and carcass characteristics compared to other breeds. Despite the breed’s popularity, its developmental history, genetic diversity, and genetic relationships with other pig breeds remain largely unknown. Therefore, the primary objective of this study was to investigate population structure and genetic diversity of Duroc subpopulations from Europe, North America, and Australia, and of other pig breeds.

**Results:**

The studied pig populations were differentiated into five subgroups(European and North American Durocs, Australian Durocs, Asian-Pacific pig breeds, and two other breed groups [OBP1 and OBP2]), consistent with their geographical origins, as revealed by population structure analyses. The estimated effective population size (Ne) of Duroc subpopulations ranged from 17 to 47, while the Ne for the combined Duroc subpopulations was 172. A total of 140,713 runs of homozygosity (ROHs) were identified across all individuals, with 98,039 ROHs in Durocs and 42,674 in other pig breeds. Durocs had a greater number and proportion of longer ROHs (> 8 Mb) compared to other pig breeds. The ROH-based inbreeding (F_ROH_) values were significantly greater in Durocs than in most of the other breeds, indicating the need for better management of genetic diversity in the breed. We observed strong correlations (> 0.65) between different inbreeding metrics in all the studied pig populations. A total of 43, 18, 27, 37, and 20 candidate genes were identified in the ROH islands for European and North American Durocs, Australian Durocs, Asian-Pacific pigs, OBP1, and OBP2 pigs, respectively. The significant KEGG pathways were mainly related to growth, metabolism, immune system, cellular processes, and signal transduction.

**Conclusions:**

Significant differences exist in genetic diversity, population structure, and ancestry within Duroc subpopulations and between Duroc and other pig breeds. The observed inbreeding levels in Duroc subpopulations indicate the need for better management of genetic diversity within the breed. Functional enrichment analyses of shared ROH islands provide new insights into biological pathways shaped by selection decisions in the past decades, especially those related to the immune system and energy metabolism.

**Supplementary Information:**

The online version contains supplementary material available at 10.1186/s12711-025-01017-6.

## Background

**Fig. 1:**
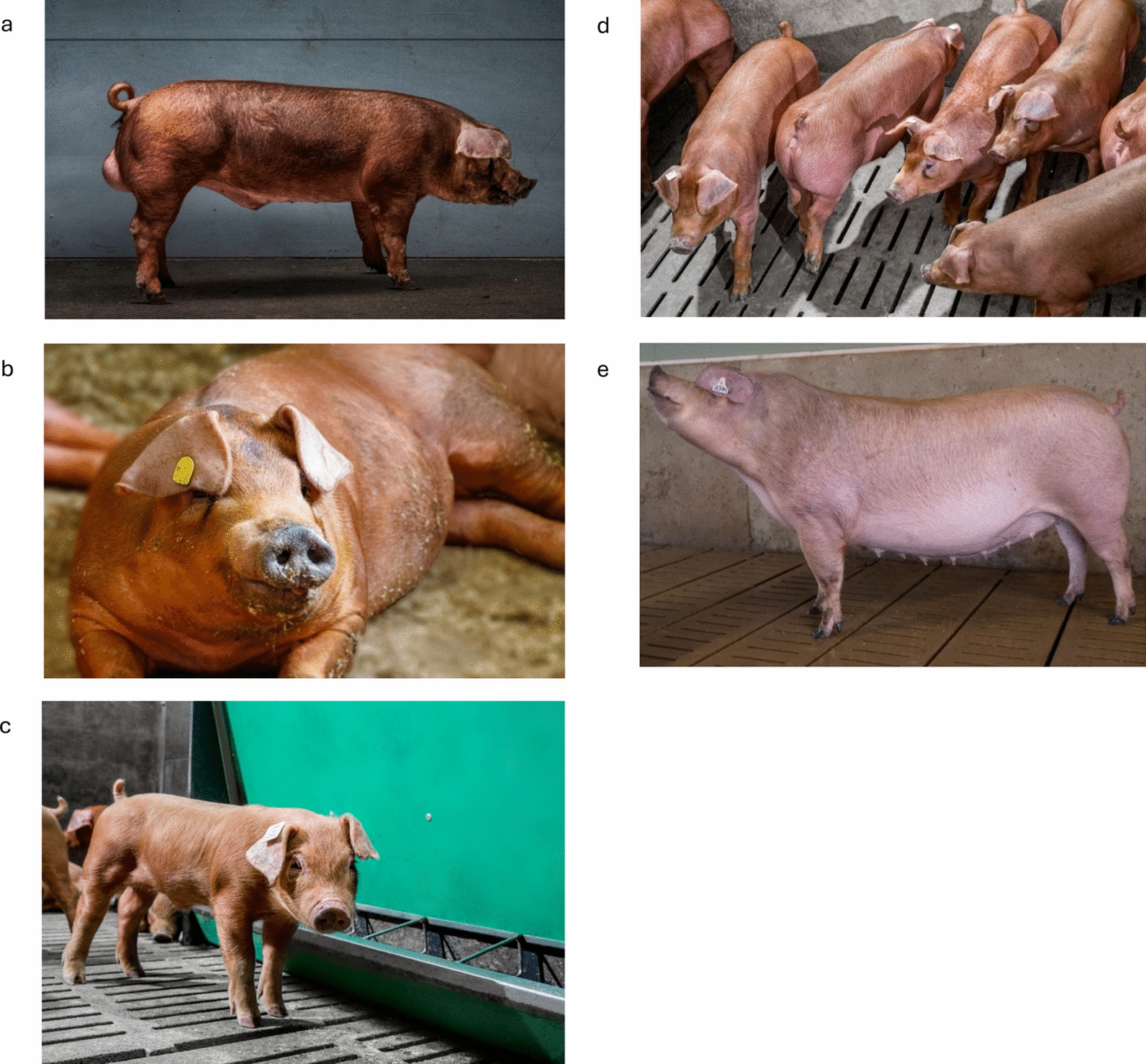
Figures of Duroc pigs. **a** and **b** European Durocs (Photo credits: Topigs Norsvin); **c** a North American Duroc piglet (Photo credits: Hendrix Genetics); **d** North American Duroc gilts (Photo credits: Hendrix Genetics); and, **e** an Australian Duroc sow

Duroc is one of the most popular terminal sire pig breeds in the world due to its high growth rate, meat quality, feed efficiency, and carcass characteristics [1]. The Duroc breed (Fig. 1) was developed in the United States in the early to mid-1800s, particularly in the areas around New York and New Jersey [2]. However, the origin of Durocs and the breeds that contributed to their formation are still unknown. The Duroc breed provides an interesting model for evaluating the genetic diversity of *Sus scrofa*. Previous scientific literature has consistently shown them to be unique when compared to other pig breeds. Furthermore, both principal component (PCA) and admixture analyses by Ding et al. [3] indicate that the Duroc breed is distinct from European and Asian pig breeds. The history of the Duroc breed can be traced back to 1812, when “Red Hogs” (pigs with a reddish coat color and undefined breed) were being bred in New York and New Jersey. The modern Duroc is believed to have been developed between 1822 and 1877 by combining the Old Duroc from New York and the Jersey Red (so named in 1857) from New Jersey. The American Duroc-Jersey Association was formed in 1883 [4]. In the early formation of the breed, Durocs were known to gain weight quickly and to have relatively large litters [5]. The deeper origins of Duroc progenitors are, however, not clear. There are reports of red pigs being imported from the UK, perhaps a Red Berkshire [6]. However, it is also known that, similar to the trade routes for goats proposed by Paim et al. [7], red hogs from the Iberian Peninsula and Africa, may have played a role in breed formation of the Duroc.

Over the past century, Durocs have been exported from the United States to many other countries and bred into various lines with distinct characteristics through continuous selection and breeding programs. However, many questions still remain regarding the development history of the Duroc breed, such as its genetic diversity and its genetic relationships with other pig breeds. Previous research has shown that genetic diversity in the Duroc breed has decreased over the past decade in China [16] and elsewhere. Wang et al. [8] reported that American Duroc pigs exhibit greater genetic diversity and lower inbreeding levels compared to Canadian Durocs. These findings indicate that there may be genetic differences within the breed due to various selection intensities and breeding goals across geographical regions and breeding companies, as well as potential introgressions from other breeds in some Duroc subpopulations. Therefore, it is important to quantify the genetic structure and variability within and between Duroc subpopulations and other pig breeds. Such an evaluation is expected to contribute to our understanding of genetic diversity in the Duroc breed and to the development of sustainable breeding strategies and the potential exchange of genetic material across subpopulations.

Due to the breed’s popularity as terminal sire in commercial crossbreeding schemes, Duroc pigs have undergone intensive selection for key breeding goals based on terminal sire indexes focusing on postweaning performance, carcass characteristics, feed efficiency, and meat quality [9, 10]. The most commonly evaluated traits in Duroc pigs include average daily gain, feed conversion ratio, backfat thickness, and intramuscular fat [8]. Evaluating the inbreeding level and maintaining sufficient genetic diversity in each (sub)population is essential for long-term sustainable genetic improvement. Accumulation of inbreeding can increase the likelihood of genetic drift and the appearance of harmful recessive mutations [11]. Furthermore, decreased genetic diversity can undermine selection progress for any trait in a breeding program [12]. This is particularly important for enhancing their fitness and adaptation to face global warming conditions [13]. Moreover, understanding inbreeding levels and overall genetic diversity within the Duroc breed is essential for informing future breeding decisions and could contribute to long-term conservation strategies, including the use of national gene banks [14, 15].

The availability of large-scale genomic information allows us to evaluate runs of homozygosity (ROH) for individuals and populations. ROH has been widely used to investigate evolutionary history and the inbreeding levels in populations by examining different patterns of ROH length and distribution in the genome [17]. For instance, Shi et al. [18] reported twelve ROH islands harboring genes associated with reproduction, muscle development, fat deposition, and adaptation in Yorkshire pigs. However, a comprehensive comparison of ROH among Durocs and other breeds has not yet been performed.

In this study, we systematically compared the genome of domestic Duroc pigs from different geographic origins and Duroc subpopulations from various corporate lines and smaller Duroc breeders. Our main objectives were to: (1) assess the within- and between-population genetic diversity of Duroc pigs from Europe, North America, and Australia, in comparison with other 13 pig breed populations; (2) quantify the genomic inbreeding level of each (sub)population; and, (3) identify shared ROH islands between subgroups and fixation index (F_ST_) regions between Duroc and Yorkshire (a maternal line breed) populations.

## Material and methods

### Populations studied

Data from 1,858 pigs were used in this study, including 1,409 Duroc pigs and 449 pigs from other breeds (abbreviated as “OBPs”, including Berkshire, Chester White, Fengjing, Hampshire, Hawaiian, Hereford, Landrace, Large Black, Meishan, Minzhu, Spotted, Tamworth, and Yorkshire breeds; Table 1). Thirteen Duroc subpopulations were sampled. Twelve of which were from corporate nucleus lines, and one (Duroc9) was a White Duroc population, which is a composite population developed based on Durocs and maternal line breeds (e.g., Yorkshire). Although the term “White Duroc” is commercially used, to our knowledge, there is no official breed definition for this composite population. The corporate lines were from Europe (n = 1, Duroc13), Australia (n = 3, Duroc8, Duroc10, Duroc11), Canada (n = 3, Duroc4, Duroc5, Duroc6), and the United States of America (n = 5, Duroc1, Duroc2, Duroc3, Duroc7, Duroc12). However, some of these subpopulations are owned by large multinational companies that have nucleus herds in multiple countries or continents. One Duroc subpopulation (Duroc3) was curated by the USDA’s National Animal Germplasm Program (NAGP) as part of their work to conserve livestock genetic resources. The NAGP animals were broadly sampled from traditional purebred Duroc breeders in North America. Data on animals from other pig breeds included in the study were also provided by the NAGP swine collection. However, some of the large populations may have been underrepresented based on the sampling strategies used, especially the largest and most common breeds in certain geographical regions (e.g., Danish Landrace and Chinese Meishan).

**Table 1:** Descriptive statistics and genetic diversity estimates for each pig population

Group	Breed	Region	N^a^	SNP N^b^	MAF^c^	Ho^d^	He^e^
Duroc	Duroc1	North America	66	32,992	0.276	0.374	0.366
	Duroc2	North America	100	33,454	0.269	0.364	0.360
	Duroc3	North America	76	44,513	0.282	0.365	0.372
	Duroc4	North America	100	39,644	0.273	0.368	0.363
	Duroc5	North America	100	35,503	0.282	0.371	0.371
	Duroc6	North America	100	31,586	0.270	0.363	0.360
	Duroc7	North America	298	18,331	0.323	0.394	0.414
	Duroc8	Australia	88	32,093	0.281	0.388	0.372
	Duroc9 (White Duroc)	North America	85	28,679	0.270	0.376	0.360
	Duroc10	Australia	96	32,424	0.288	0.388	0.378
	Duroc11	Australia	100	29,939	0.296	0.392	0.385
	Duroc12	North America	100	32,443	0.274	0.368	0.365
	Duroc13	Europe	100	31,921	0.266	0.365	0.356
Other breeds	Berkshire	UK	46	46,172	0.266	0.347	0.355
	Chester White	USA	27	51,930	0.285	0.376	0.375
	Fengjing	China	19	28,798	0.237	0.359	0.325
	Hampshire	UK	40	44,020	0.261	0.357	0.350
	Hawaiian	USA-Hawaii	35	43,432	0.293	0.325	0.382
	Hereford	USA	22	44,106	0.283	0.380	0.372
	Landrace	Denmark	35	51,944	0.304	0.398	0.392
	Large Black	UK	8	40,536	0.263	0.389	0.351
	Meishan	China	52	27,015	0.239	0.312	0.328
	Minzhu	China	19	48,598	0.267	0.358	0.357
	Spotted	USA	10	49,989	0.271	0.368	0.358
	Tamworth	UK	16	48,481	0.281	0.381	0.371
	Yorkshire	UK	120	51,377	0.296	0.375	0.385

^a^N: Number of studied animals

^b^SNP N: Number of single nucleotide polymorphisms (SNP) after quality control

^c^MAF: Minor allele frequency

^d^Ho: Observed homozygosity

^e^He: Expected homozygosity

### SNP genotyping and quality control

Detailed information on the breeds included in this study, including the geographical regions of data collection, breed names, sample size, and number of single nucleotide polymorphisms (SNP) after quality control (QC), are presented in Table 1. The different populations were genotyped using a wide range of SNP arrays, including proprietary genotyping platforms. QC was conducted using the PLINK software v1.90 [19] based on removal of: (1) SNPs with a call rate lower than 0.95; (2) SNPs with a MAF lower than 0.05; (3) SNPs and individuals with a call rate lower than 0.90; (4) SNPs with extreme departure from Hardy–Weinberg Equilibrium (HWE; *P*-value < 10^–6^) as an indication of genotyping error; (5) SNPs with unknown or duplicated positions on the genome; and, (6) SNPs located in non-autosomal chromosomes. The SNP genome coordinates were obtained from the *Sscrofa* 11.1 porcine reference genome assembly (http://useast.ensembl.org/Sus_scrofa/Info/Index). After QC, data from 1,858 individuals were kept for further analyses. Please note that the QC differed depending on the analysis, as explained below.

### Population structure analyses

Population structure was evaluated by performing PCA using 3,221 SNPs shared among all populations (after QC). PCA was conducted using the PLINK software v1.90 [19]on all populations (n = 1,858) and also exclusively on the Duroc subpopulations. The R software [20] was used for plotting the PCA results, which were based on a variance-standardized genomic relationship matrix. Ancestry estimation was performed using shared genomic data (3,221 SNPs after QC) among all populations and different numbers of ancestral populations were tested for the Duroc subpopulations (K = 2 to 5) and for all populations (K = 2 to 17) using the ADMIXTURE software v1.2 [21]. The optimal K value was determined based on the lowest cross-validation (CV) error and used for plotting the results (see Additional file 1, Fig. S1). The genetic distance matrix of all studied samples was calculated using the PLINK software v1.90 [19]. A neighbor-joining (NJ) phylogenetic tree was constructed using the FastME package v2.0 [22] and plotted using the Chiplot v2.6 package (www.chiplot.online/tvbot.html [23]).

Linkage disequilibrium (LD, ${r}^{2}$) was calculated for each population using full, population-specific genotype information (after QC, please see in Table 1) and PLINK software v1.90 [19]. In addition, effective population size (Ne) was estimated as [24]:

1 $${N}_{E(t)}=\frac{1}{\left(4f\left({c}_{t}\right)\right)}\left(\frac{1}{E\left[\left.{r}_{adj}^{2}\right|{c}_{t}\right]}-\alpha \right)$$

Here, ${N}_{E(t)}$ is the Ne estimated t generations in the past, ${c}_{t}$ is the recombination rate for a specific physical distance between SNP markers t generations in the past, $\alpha$ is the probability of the occurrence of mutation, and ${r}_{adj}^{2}$ is the LD value calculated based on the correlation between two alleles in from a pair of loci as [24]:

2 $${r}_{adj}\left({p}_{a},{p}_{b},{p}_{ab}\right)=\frac{{({p}_{ab}-{p}_{a}{p}_{b})}^{2}}{{p}_{a}(1-{p}_{a}){p}_{b}(1-{p}_{b})},$$

where ${p}_{a}$ is the frequency of allele $a$, ${p}_{b}$ is the frequency of allele $b$, and ${p}_{ab}$ is the haplotype frequency with allele $a$ at the first locus and allele $b$ at the second locus. We estimated Ne at different numbers of past generations (ranging from 25 to 100), for each population and for the whole Duroc population.

### Identification of runs of homozygosity

Runs of homozygosity were detected in each population using the PLINK v1.90 software [19]. For ROH analyses, each population was analyzed separately using its full, population-specific SNP set, and the genotype data was not filtered for low MAF. ROH were identified using the following [18], (1) minimum ROH length of 500 kb; (2) a minimum of 15 consecutive SNPs; (3) a maximum gap between consecutive SNPs of 500 kb; (4) a minimum density of one SNP per 75 kb; (5) a sliding window of 50 SNPs across the genome that moved one SNP at a time; and, (6) a maximum of five missing genotypes and one heterozygous genotype. Five classes of ROHs were defined based on their length: < 2 Mb, 2 – 4 Mb, 4 – 8 Mb, 8 – 16 Mb, and > 16 Mb. ROH islands were identified based on the per-SNP ROH incidence, calculated as the percentage of individuals for which the SNP was included in a ROH. A dual-threshold approach was used to define relevant SNPs, which were required to satisfy two criteria: (1) an incidence within the top 1% quantile (99th percentile) of that population’s empirical distribution [25], and (2) an incidence of at least 35%. Finally, candidate SNPs that satisfied both conditions and that were separated by less than 100 kb were merged to form contiguous ROH island regions.

The detectRUNs [26] and GenomicRanges [27] R packages were used to detect shared ROH islands in five groups of subpopulations that were identified based on PCA and phylogenetic tree analyses. These included European and North American Durocs, Australian Durocs (Duroc9, Duroc10, and Duroc11), Asian-Pacific breeds (Meishan, Minzhu, Fengjing, and Hawaiian pigs), OBP1 (Tamworth and Hereford), and OBP2 (Berkshire, ChesterWhite, Hampshire, Landrace, Large Black, Spotted, and Yorkshire). The shared ROH islands were used to retrieve positional candidate genes, whichwere annotated based on the latest genome reference *Sscrofa 11.1* assembly (http://useast.ensembl.org/Sus_scrofa/Info/Index). Gene Ontology (GO) terms [28] and Kyoto Encyclopedia of Genes and Genomes (KEGG) [29] enrichment analyses were conducted for the candidate genes using the ClusterProfiler R package [30].

### Genomic inbreeding coefficients

Ten metrics of genomic inbreeding coefficients ($\text{F}$) were calculated using the PLINK software v1.90 [19], including:

- ${\text{F}}_{\text{GRM}},$ genomic inbreeding coefficients based on the genomic relationship (**G**) matrix, which was calculated as [31]:
  3 $${\text{F}}_{{\text{GRM}}_{\text{i}}}={\mathbf{G}}_{\mathbf{i}\mathbf{i}}-1,$$
  where ${\mathbf{G}}_{\mathbf{i}\mathbf{i}}$ is the* i*th **G** matrix diagonal element for individual $\text{i}$
- ${\text{F}}_{\text{ROH}},$ genomic inbreeding coefficients based on all ROH segments. Individual ${\text{F}}_{\text{ROH}}$ (i.e., proportion of the genome covered by ROH) was calculated as:
  4 $${\text{F}}_{{{\text{ROH}}}} = \frac{{\mathop \sum \nolimits_{{\text{i}}} {\text{L}}_{{{\text{ROH}}_{{\text{i}}} }} }}{{{\text{L}}_{{{\text{auto}}}} }},$$
  where ${\text{L}}_{{\text{ROH}}_{\text{I}}}$ is the length of all ${\text{ROH}}_{\text{I}}$ of individual $\text{i}$ and ${\text{L}}_{\text{auto}}$ is the autosomal genome length covered by the SNPs that passed the QC. The ${\text{F}}_{\text{ROH}}$ for each individual was calculated including all ROH segments (${\text{F}}_{\text{ROH}}$), ROH segments < 2 Mb (${\text{F}}_{\text{ROH}\left(<2\text{Mb}\right)}$), as well as ROH segments ranging from 2 to 4 Mb (${\text{F}}_{\text{ROH}\left(2-4\text{Mb}\right)}$), 4-8 Mb (${\text{F}}_{\text{ROH}\left(4-8\text{Mb}\right)}$), 8-16 Mb (${\text{F}}_{\text{ROH}\left(8-16\text{Mb}\right)}$), and > 16 Mb (${\text{F}}_{\text{ROH}\left(> 16\text{Mb}\right)}$) in length.
- ${\text{F}}_{\text{HOM}1},$ genomic inbreeding based on the difference between observed and expected homozygous genotypes, calculated as [19]:
  5 $${\text{F}}_{\text{HOM}1}=\frac{{\text{H}}_{\text{exp}}-{\text{H}}_{\text{obs}}}{{\text{H}}_{\text{exp}}},$$
  where ${\text{H}}_{\text{exp}}$ and ${\text{H}}_{\text{obs}}$ are the expected and observed values, respectively, for homozygous genotypes.
- d) ${\text{F}}_{\text{HOM}2,}$ genomic inbreeding based on homozygous genotype, which was calculated as:
  6 $${\text{F}}_{\text{HOM}2}=1-\frac{{\text{x}}_{\text{i}}*\left(2-{\text{x}}_{\text{i}}\right)}{{2\text{p}}_{\text{i}}\left(1-{\text{p}}_{\text{i}}\right)},$$
  where ${\text{x}}_{\text{i}}$ is the number of reference allele copies at the ith SNP and ${\text{p}}_{\text{i}}$ is the reference allele frequency in the population.
- ${\text{F}}_{\text{UNI}},$ genomic inbreeding coefficients based on uniting gametes, calculated as [32]:
  7 $${\text{F}}_{\text{UNI}}=\frac{{\text{x}}_{1}^{2}-(1+2{\text{p}}_{\text{i}}{)*\text{x}}_{\text{i}}+2{\text{p}}_{\text{i}}^{2}}{2{\text{p}}_{\text{i}}(1-{\text{p}}_{\text{i}})}$$

Pearson’s correlations were calculated between all pairs of genomic inbreeding coefficients. A heatmap was created to visualize the inbreeding coefficient correlations using the ggplot2 R package [33].

### Selective signals

To more accurately identify potential selective signals (selection sweeps) between terminal sire (Duroc) and maternal (Yorkshire) lines, we used a sliding-window approach (100-kb windows sliding in 10-kb steps) to quantify the levels of genetic differentiation (F_ST_) between Duroc (European and North American Durocs and Australian Durocs) and Yorkshire pigs using VCFtools (v.0.1.14, [34]). The full (population-specific) genotype information (after QC, Table 1) was used for these analyses. Genomic windows that were in the top 1% based on F_ST_ were considered as selection sweeps.

## Results

### Genetic diversity

Descriptive statistics and genetic diversity estimates for each population, including geographical regions of data collection, breed names, sample size, number of SNPs after QC, average MAF, observed heterozygosity (Ho), and expected heterozygosity (H_E_) are presented in Table 1. The MAF ranged from 0.27 (Duroc13) to 0.32 (Duroc7) and from 0.24 (Fengjing) to 0.30 (Landrace) in Durocs and OBPs, respectively. In Durocs, Ho and He ranged from 0.36 (Duroc6) to 0.39 (Duroc7) and from 0.36 (Duroc13) to 0.41 (Duroc7), respectively. In OBPs, Ho and He ranged from 0.31 (Meishan) to 0.40 (Landrace) and from 0.33 (Fengjing) to 0.39 (Landrace) and, respectively. Two pig breeds (Meishan and Fengjing pigs) had lower MAF and H_E_ indicating that the sampled Chinese indigenous pig breeds had relatively low levels of genetic diversity (Table 1). Most populations in our study exhibited similar levels of H_E_ and Ho. However, Fengjing pigs had higher Ho (0.36) than H_E_ (0.33), while Hawaiian pigs had greater H_E_ (0.38) than Ho (0.33).

### Population structure analyses

**Fig. 2:**
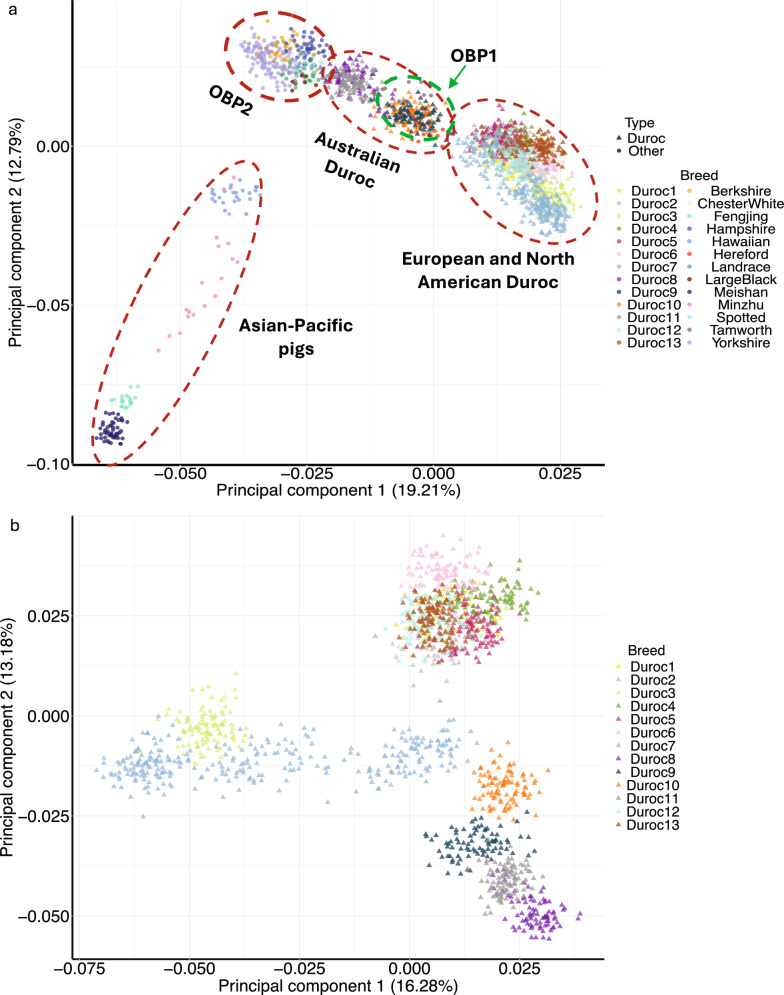
Global genetic structure of various Duroc sub-populations and other pig breeds

**Fig. 3:**
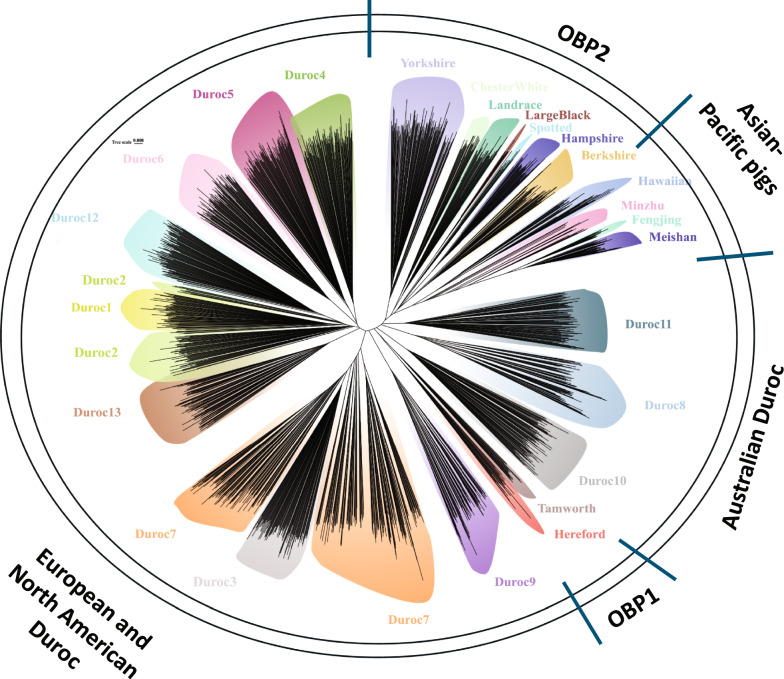
Neighbor-joining phylogenetic tree of 1835 individuals from Duroc sub-populations and other pig breeds

The genetic relationships among pig populations were examined using PCA (Fig. 2a and b) and phylogenetic trees (Fig. 3). The first three principal components explained 19.2%, 12.8%, and 10.4% of the total variation for all Durocs, respectively. The first two PCs differentiated the European and North American Durocs, Australian Durocs, Asia–Pacific pigs, OBP1 (Tamworth and Hereford), and OBP2 (Berkshire, Chester White, Hampshire, Landrace, Large Black, Spotted, and Yorkshire) subgroups (Fig. 2a). The Duroc3 and Duroc7 clustered together based on the Fig. 2b, especially compared to other Duroc populations. The same five clades (see Fig. 3) were observed in the NJ tree. Clade I consisted of all Durocs from North America and Europe, Clade II was formed by Tamworth and Hereford, which are phenotypically similar to the Duroc breed, Clade III consisted of Durocs from Australia, Clade IV included Chinese indigenous pigs and Hawaiian pigs, and Clade V contained the remaining breeds that originated in Europe. OBP1 pigs (Hereford and Tamworth) occupied intermediate positions between the two major clades—European and North American Durocs, and Australian Durocs. This pattern was observed based on both PCA (Fig. 2) and NJ phylogenetic tree (Fig. 3) results.

The European and North American Durocs clade in the phylogenetic tree included four main branches: (i) Duroc9, the White Duroc population, is a composite population of Duroc and white pig breeds (e.g., Yorkshire or Landrace). Both of these breeds are commonly used in commercial breeding for their desirable traits; (ii) Duroc7 and Duroc3, with Duroc7 further subdividing into three sub-branches; (iii) Duroc2, which was divided into two parts; and, (iv) Duroc4, Duroc5, and Duroc6, which clustered together.

**Fig. 4:**
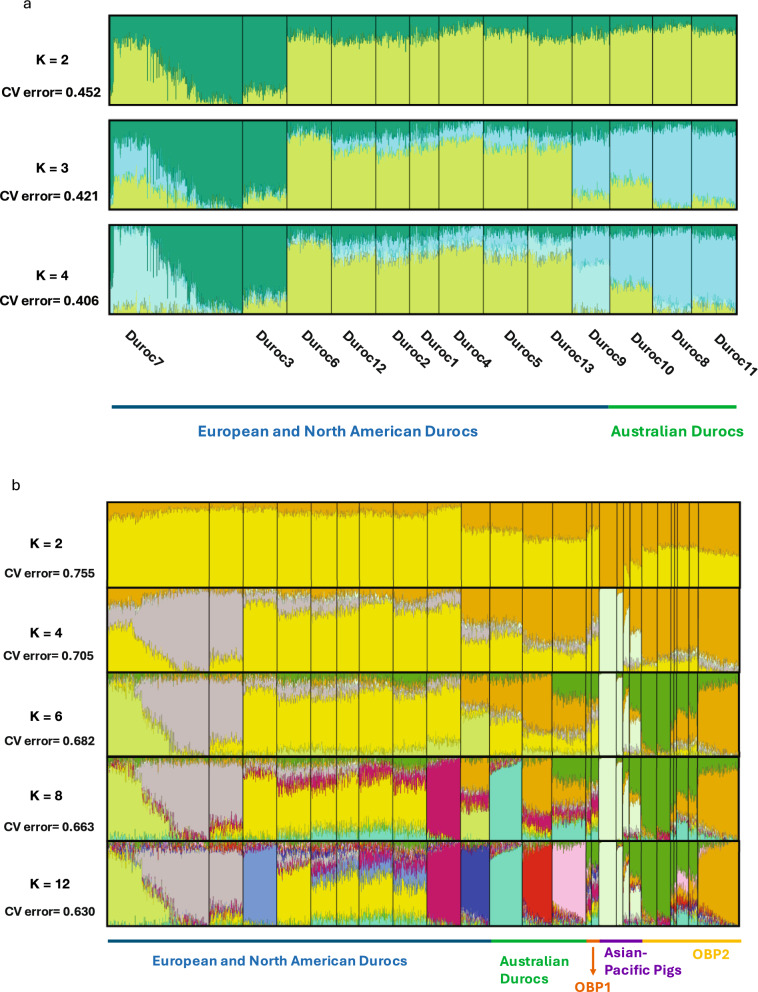
Admixture analysis **a)** in Duroc sub-populations (K = 2 to 4); and **b)** in all pig populations (K = 4 to 15). Each vertical bar stands for an individual and each color represent a different ancestral population. In Fig. 4b, the pig populations are Duroc7, Duroc3, Duroc6, Duroc12, Duroc2, Duroc1, Duroc4, Duroc5, Duroc13, Duroc9, Duroc10, Duroc8, Duroc11, Tamworth, Hereford, Meishan, Fengjing, Minzhu, Hawaiian, Berkshire, Hampshire, Spotted, Landrace, Chester White, and Yorkshire, respectively.

For the combined Duroc populations, ADMIXTURE was run for K values ranging from 2 to 5 (Fig. 4a for K from 2 to 4). The lowest CV error was found for K = 4, which represents the most probable number of ancestral populations in Durocs. At K = 2, the majority of Duroc populations had relatively similar proportions of the two clusters, however two populations (Duroc7 and Duroc3) did not exhibit this pattern, consistent with the results observed based on the PCA (Fig. 2b). At K = 3, the Australian populations differed from the European and North American populations, except for Duroc3, Duroc7, and Duroc9. At K = 4, the proportion assignments to the clusters differed globally (e.g., Duroc7 and Duroc3, Australian Duroc group, and the rest of the Duroc populations).

To compare Durocs and other pig breeds, K values from 2 to 17 (Fig. 4b) were tested for all pig populations included in the study. However, for K = 13 to 17, the CV error values decreased by less than 1% per unit increase in K. Therefore, only results for K values of 2, 4, 6, 8, 12, and 15 were plotted. At K = 4, European and North American Durocs, Australian Durocs, OBP1, and OBP2 were more similar to each other. At K = 2, 4, 6, and 8, the Duroc9 White Duroc population (located at the extreme right of the European and North American Duroc population cluster) showed a more similar ancestral background with Australian Durocs, a pattern also observed in the PCA plot. This may indicate that both White Duroc and Australian Durocs have genetic contributions from similar other breeds (e.g., Yorkshire). At K = 12, we observed greater variability within European and North American Durocs.

**Fig. 5:**
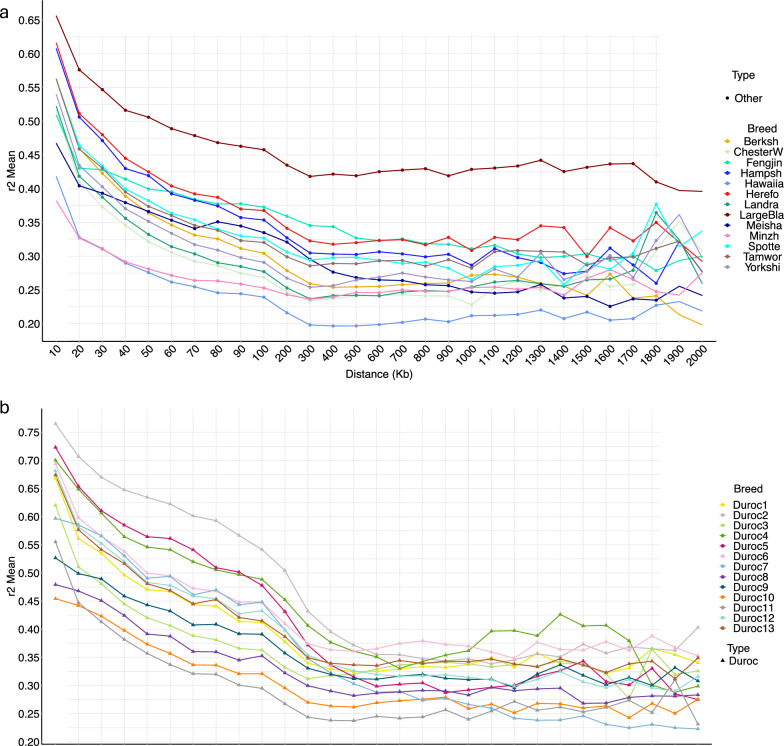
Linkage disequilibrium (LD; measured as r^2^) of different pig populations. **a)** LD of selected pig breeds; **b)** LD of Duroc sub-populations

To compare the extent of LD across all studied populations, mean ${r}^{2}$ was calculated for each 100 kb window and the LD decay based on physical distance between SNP pairs up to 2,000 kb was plotted (Fig. 5a and b). European and North American Durocs had relatively higher LD levels compared to Australian Durocs (Fig. 5b) but these differences decreased as the distance between markers increased and approached background LD levels. The latter refers to average level of LD that is more stable across the genome in a population, at 500 kb distance in all the Duroc populations, with an average ${r}^{2}$= 0.316. However, most OBP populations reached their background LD levels at 300 kb (see Fig. 5a). More rapid LD decay was observed for the other breeds than for the Duroc subpopulations.

**Fig. 6:**
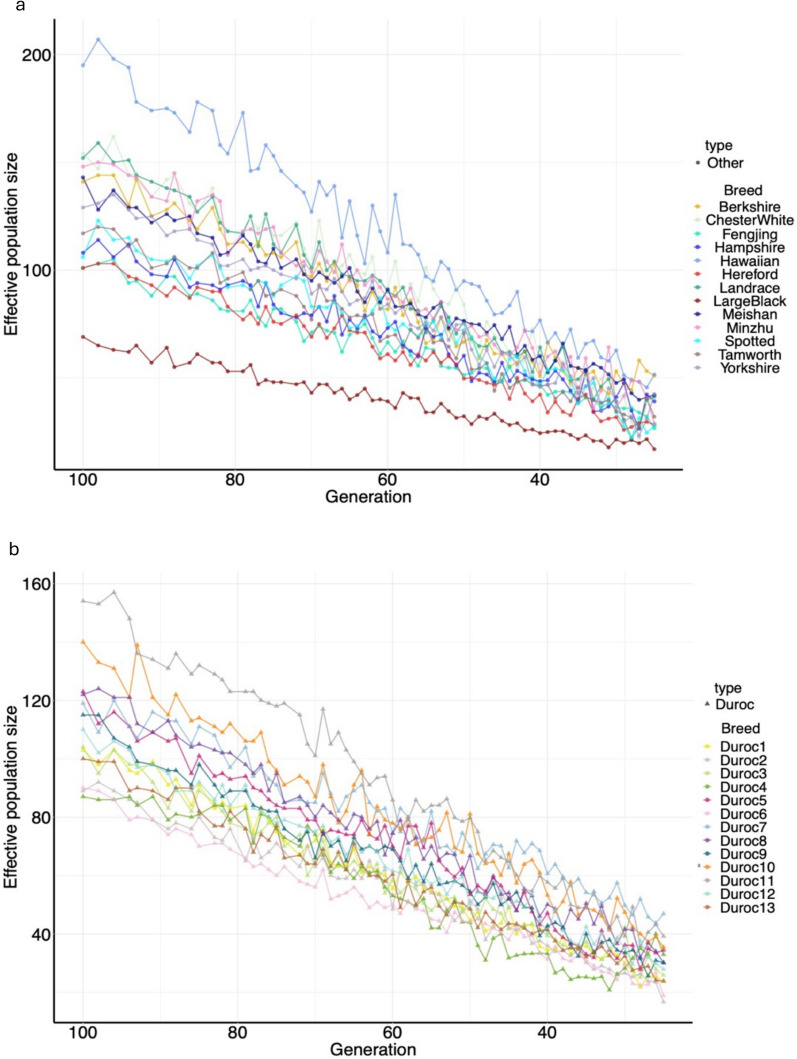
Effective population size (Ne) of different pig populations. **a)** Ne of selected pig breeds; **b)** Ne of each Duroc sub-population

We also estimate the Ne of all studied populations over their past 25 to 100 generations (Fig. 6 and see Additional file 3, Table S1). The estimated Ne (172) for the whole Duroc population indicates substantial genetic variability across sub-populations. The estimated Ne for Duroc subpopulations at the most recent evaluated generation (generation 25) ranged from 17 (Duroc2) to 47 (Duroc7). Australian Durocs had relatively high Ne (30 for Duroc8 to 39 for Duroc11). Hawaiian pigs had the highest Ne estimate (52) in the most recent generation, while Large Black pigs had the lowest estimate of Ne (17).

Among the ten genomic inbreeding coefficients evaluated, F_ROH_ consistently had the highest estimates across all populations (Table 2). The Duroc3 population showed the greatest inbreeding based on F_ROH_ (0.362), not only among Durocs but across all populations studied. In contrast, the indigenous Fengjing population exhibited the lowest average genomic inbreeding based on ${F}_{HOM1}$ (-0.104), ${F}_{GRM}$ (-0.086), ${F}_{HOM2}$ (-0.086), and ${F}_{UNI}$ (-0.086), except for ${F}_{ROH}$ and its derived metrics.

**Table 2:** Measures of genomic inbreeding coefficients in Duroc subpopulations and other pig breeds

Group	Breed	FHOM1^a^	FGRM^b^	FHOM2^c^	FUNI^d^	FROH^e^	< 2Mb^f^	2− 4Mb^g^	4− 8Mb^h^	8− 16Mb^i^	> 16Mb^j^
Duroc	Duroc1	0.190	0.098	0.155	0.154	0.098	0.005	0.029	0.034	0.022	0.008
	Duroc2	− 0.011	− 0.011	− 0.011	− 0.011	0.262	0.025	0.052	0.064	0.066	0.088
	Duroc3	0.030	0.025	0.030	0.027	0.362	0.019	0.059	0.088	0.097	0.100
	Duroc4	− 0.015	− 0.014	− 0.014	− 0.014	0.305	0.006	0.063	0.085	0.072	0.080
	Duroc5	− 0.001	− 0.001	− 0.001	− 0.001	0.149	0.010	0.036	0.050	0.037	0.016
	Duroc6	− 0.009	− 0.010	− 0.010	− 0.010	0.249	0.011	0.053	0.064	0.068	0.054
	Duroc7	0.049	0.044	0.044	0.044	0.035	0.002	0.012	0.013	0.006	0.001
	Duroc8	− 0.043	− 0.042	− 0.042	− 0.042	0.086	0.003	0.017	0.026	0.023	0.017
	Duroc9 (White Duroc)	− 0.044	− 0.042	− 0.042	− 0.042	0.086	0.003	0.020	0.028	0.025	0.011
	Duroc10	− 0.026	− 0.027	− 0.027	− 0.027	0.083	0.004	0.021	0.028	0.018	0.012
	Duroc11	− 0.018	− 0.018	− 0.018	− 0.018	0.120	0.008	0.028	0.035	0.032	0.017
	Duroc12	− 0.007	− 0.006	− 0.006	− 0.006	0.066	0.004	0.020	0.026	0.014	0.002
	Duroc13	− 0.025	− 0.024	− 0.024	− 0.024	0.228	0.012	0.054	0.062	0.058	0.043
Other Breeds	Berkshire	0.030	− 0.032	0.050	0.009	0.222	0.028	0.057	0.064	0.061	0.078
	ChesterWhite	− 0.004	− 0.005	− 0.005	− 0.005	0.141	0.014	0.029	0.029	0.031	0.038
	Fengjing	− 0.104	− 0.086	− 0.086	− 0.086	0.203	0.019	0.049	0.040	0.031	0.064
	Hampshire	− 0.019	− 0.021	− 0.021	− 0.021	0.236	0.023	0.050	0.060	0.047	0.057
	Hawaiian	0.105	0.105	0.104	0.105	0.168	0.018	0.038	0.042	0.033	0.035
	Hereford	− 0.020	− 0.020	− 0.020	− 0.020	0.232	0.019	0.040	0.049	0.053	0.071
	Landrace	− 0.012	− 0.011	− 0.011	− 0.011	0.114	0.014	0.029	0.030	0.025	0.017
	LargeBlack	− 0.093	− 0.090	− 0.090	− 0.090	0.246	0.019	0.040	0.048	0.054	0.085
	Meishan	0.047	0.044	0.044	0.044	0.273	0.019	0.041	0.056	0.051	0.105
	Minzhu	− 0.004	0.002	0.002	0.002	0.106	0.009	0.020	0.018	0.015	0.043
	Spotted	− 0.028	− 0.031	− 0.031	− 0.031	0.166	0.014	0.030	0.033	0.031	0.058
	Tamworth	− 0.028	− 0.027	− 0.027	− 0.027	0.173	0.012	0.027	0.030	0.043	0.062
	Yorkshire	0.030	0.042	0.032	0.037	0.183	0.019	0.036	0.040	0.041	0.051

^a^FHOM1: Genomic inbreeding based on homozygous genotypes observed and expected

^b^FGRM: Genomic inbreeding coefficients based on the diagonal elements of the genomic relationship matrix

^c^FHOM2: Genomic inbreeding based on homozygous genotype

^d^FUNI: Genomic inbreeding coefficients based on the uniting gametes method

^e^FROH: Genomic inbreeding coefficients based on runs of homozygosity (ROH)

^f^ < 2 Mb: Genomic inbreeding coefficients based on ROHs shorter than 2 Mb in length

^g^2-4 Mb: Genomic inbreeding coefficients based on ROHs ranging from 2 to 4 Mb in length

^h^4-8 Mb: Genomic inbreeding coefficients based on ROHs ranging from 4 to 8 Mb in length

^i^8-16 Mb: Genomic inbreeding coefficients based on ROHs ranging from 8 to 16 Mb in length

^j^ > 16 Mb: Genomic inbreeding coefficients based on ROHs greater than 16 Mb in length

**Fig. 7:**
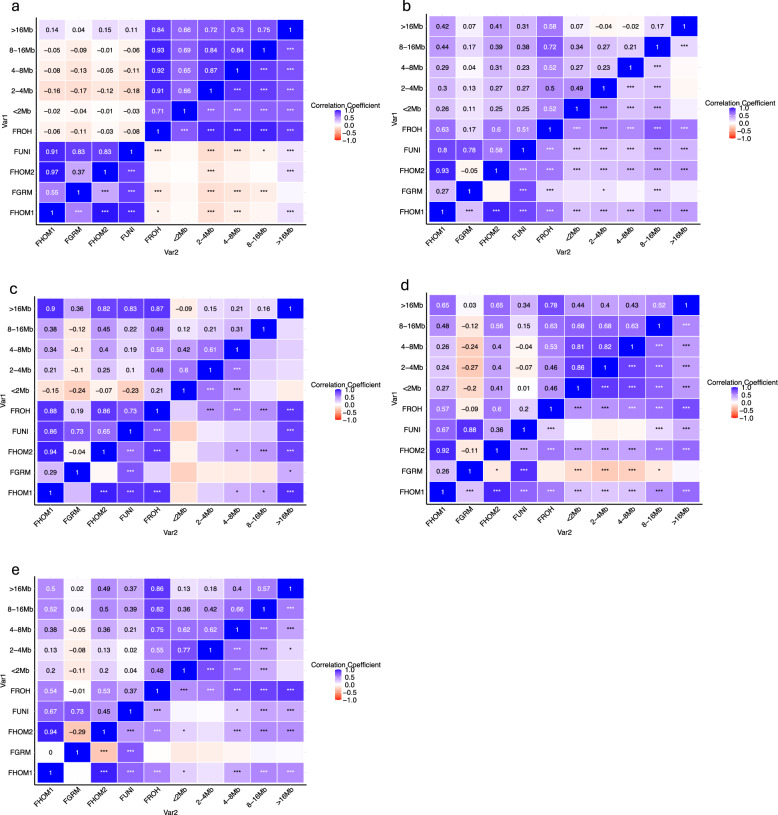
Pearson’s correlation coefficients for pairs of genomic inbreeding coefficients for the five subgroups of pigs. **a)** European and North American Durocs; **b)** Australian Durocs; **c)** OBP1; **d)** OBP2; **e)** Asian-Pacific Pigs

Pearson’s correlation coefficients among all pairs of inbreeding coefficients for each subgroup (European and North American Durocs, Australian Durocs, OBP1, OBP2, and Asian-Pacific pigs) are presented in Fig. 7. Strong correlations (> 0.67) were found for all subgroups between ${F}_{HOM1}$ and ${F}_{UNI}$, ${F}_{HOM1}$ and ${F}_{HOM2}$, and ${F}_{GRM}$ and ${F}_{UNI}$. In the European and North American Duroc populations, ${F}_{ROH}$ had zero to negative correlations (0 to − 0.11) with ${F}_{UNI}$, ${F}_{HOM2}$, ${F}_{GRM}$, and ${F}_{HOM1}$, which was not expected. In other subgroups, ${F}_{GRM}$ had correlations ranging from low negative to positive with ${F}_{ROH}$ (-0.09 for OBP2: to 0.18 for Australian Durocs), and consistently low to negative correlations with ${F}_{HOM2}$ (-0.29 for Asian-Pacific pigsto − 0.04 for OBP1). In Australian Durocs, ${F}_{ROH(>16Mb)}$ had nearly zero correlations with ${F}_{ROH(<2Mb)}$, ${F}_{ROH(2-4Mb)}$, ${F}_{ROH(4-8Mb)}$, and ${F}_{ROH(8-16Mb)}$. Conversely, in European and North American Durocs, positive and moderate to high correlations were observed among ${F}_{ROH(<2Mb)}$, ${F}_{ROH(2-4Mb)}$, ${F}_{ROH(4-8Mb)}$, ${F}_{ROH(8-16Mb)},$ and ${F}_{ROH(>16Mb)}$.

### Runs of homozygosity

Table 3 summarizes the average length and range of ROH, as well as descriptive statistics of the five classes of ROH for each population. Across all populations, we identified 140,713 ROHs, with Durocs (98,039) contributing more than twice as many as OBPs (42,674). The number of ROHs varied significantly, with Duroc3 exhibiting the highest count (n = 15,648) and the Spotted population the lowest count (n = 754). The most common ROHs were 2–4 Mb in length for both Durocs (40.0%) and OBPs (34.5%), while long ROHs (> 16 Mb) were the least frequent (3.5% and 5.5%, respectively). The average ROH length was slightly greater in Durocs (5.178 ± 5.388 Mb) than in OBPs (4.975 ± 6.795 Mb).

**Table 3:** Average length and range of runs of homozygosity (ROH) and descriptive statistics of the five classes of ROHs for each pig population

Group	Breed	Average Length (Mb)		Category				
		Mean (SD)	Range	< 2 Mb	2-4 Mb	4-8 Mb	8-16 Mb	> 16 Mb
Duroc	Duroc1	4.537(3.622)	0.504–43.114	517	1565	1006	336	59
	Duroc2	4.947(6.092)	0.502–110.365	3046	3158	1932	1021	491
	Duroc3	5.671(6.434)	0.503–115.965	3662	4965	3884	2152	985
	Duroc4	6.077(6.546)	0.505–101.345	843	5357	3768	1586	750
	Duroc5	4.498(4.136)	0.506–48.411	1937	2956	2218	840	172
	Duroc6	5.525(5.787)	0.522–80.496	1688	4523	2805	1508	534
	Duroc7	4.026(2.805)	0.565–26.163	1189	2869	1764	442	50
	Duroc8	5.638(5.154)	0.547–58.699	455	1181	1029	457	167
	Duroc9 (White Duroc)	5.244(4.387)	0.530–44.302	434	1396	1020	475	97
	Duroc10	4.944(4.394)	0.506–76.639	571	1687	1160	384	130
	Duroc11	4.874(4.719)	0.512–70.464	1216	2384	1532	724	177
	Duroc12	4.183(2.820)	0.520–28.034	657	1670	1213	333	20
	Duroc13	5.136(5.072)	0.512–69.219	1836	4589	2704	1330	433
Other breeds	Berkshire	4.939(6.698)	0.545–93.633	1589	1702	959	452	252
	Chester White	4.804(6.214)	0.515–80.091	621	692	348	179	96
	Fengjing	4.719(7.336)	0.632–95.063	618	813	340	133	99
	Hampshire	4.696(5.853)	0.514–78.189	1515	1724	1048	428	213
	Hawaiian	4.348(5.116)	0.608–73.528	1104	1154	660	263	125
	Hereford	5.361(6.883)	0.502–72.028	696	754	479	260	146
	Landrace	4.032(4.731)	0.502–68.238	842	873	465	192	54
	Large Black	5.664(7.735)	0.501–102.490	255	274	168	93	61
	Meishan	5.934(8.568)	0.515–129.485	1690	1842	1289	594	450
	Minzhu	5.570(9.871)	0.537–107.029	291	322	147	65	57
	Spotted	5.405(7.698)	0.640–81.727	233	256	148	68	49
	Tamworth	5.990(8.147)	0.611–90.923	313	376	211	149	86
	Yorkshire	4.768(6.282)	0.567–115.493	3835	3713	2134	1062	555

**Fig. 8:**
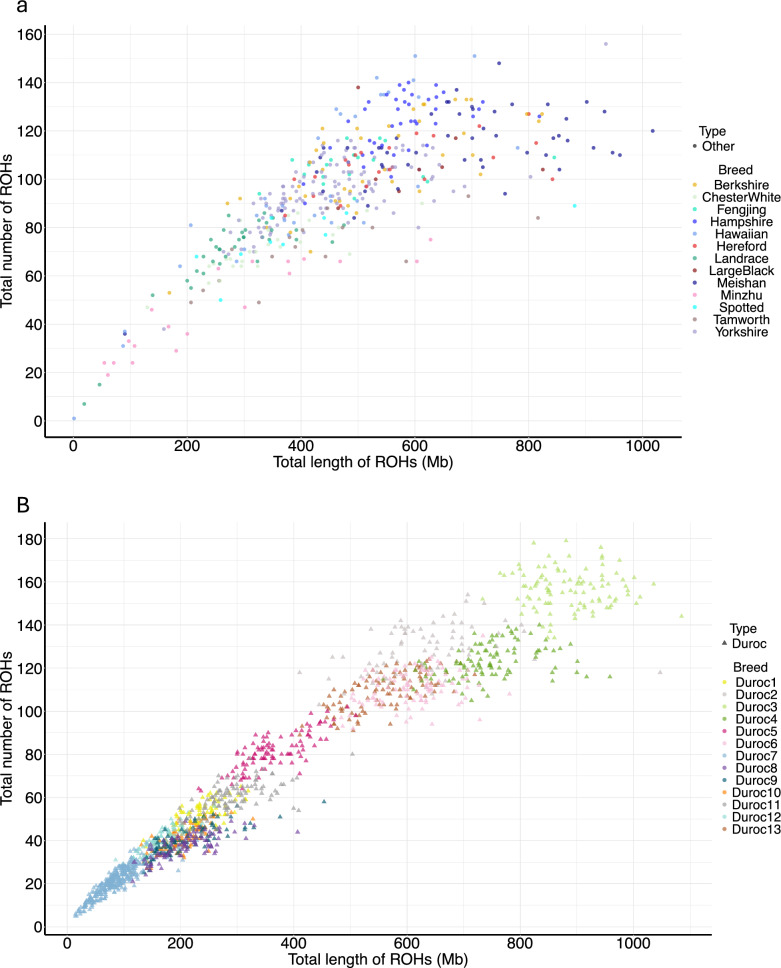
Illustration of the total number versus length of runs of homozygosity (ROH) segments of each individual of pigs. **a)** selected pig breeds; **b)** Duroc sub-populations

A key finding emerged from the geographic structuring of Durocs (Fig. 8b). The European and North American Durocs exhibited two different patterns: one group clustered near the coordinate origin and included Duroc1, Duroc5, Duroc7, and Duroc9, each carrying 1 to 80 ROH spanning 20 to 600 Mb (Fig. 8b). The other group clustered on the right-hand side, with each individual carrying 80 to 150 ROH and a total length of 500 to 1,100 Mb (Fig. 8b). Notably, the majority of Duroc3 animals had more than 120 ROH segments, covering over 800 Mb of their autosomes (nearly one-third of their genome covered by SNPs). However, it is important to emphasize that the number of SNPs available for the ROH analyses was relatively small, which can influence the length of the ROH segments, and therefore, the proportion of the genome covered by ROH segments.

### ROH islands

Identification of shared ROH regions between populations can provide valuable insights into their genetic history and selection pressures. European and North American Duroc, Australian Duroc, Asian-Pacific, OBP1, and OBP2 pigs had 4, 4, 12, 10, and 7 shared ROH islands, respectively, which were located on different chromosomes (SSC1, SSC2, SSC6, SSC7, SSC8, and SSC14). A total of 43, 18, 27, 37, and 20 genes that overlapped these regions were identified for European and North American Duroc, Australian Duroc, Asian-Pacific, OBP1, and OBP2 pigs, respectively (see Additional file 4, Table S2).

Based on the candidate genes identified, 9, 14, and 2 KEGG pathways were significantly enriched for Australian Duroc, Asian-Pacific, and OBP1 pigs, respectively (Table 4). No significant KEGG pathway was enriched for European and North American Duroc and OBP2 pigs. Enriched KEGG pathways in Australian Durocs were mainly associated with growth and tissue development, protein synthesis, and immune responses. In Asian-Pacific pigs, they were primarily linked to detoxification, environmental adaptation, and nutrient and hormone metabolism. In OBP2 pigs, enriched pathways related to reproductive system stability and long-term physiological robustness.

**Table 4:** Significantly enriched (*P* < 0.05) Kyoto Encyclopedia of Genes and Genomes (KEGG) pathways identified for the five subgroups

**Group**	**Category**	**ID**	**Description**	**P-value**
Australian Duroc	Environmental Information Processing	ssc04392	Hippo signaling pathway - multiple species	0.009
	Genetic Information Processing	ssc03008	Ribosome biogenesis in eukaryotes	0.025
	Cellular Processes	ssc04520	Adherens junction	0.03
	Organismal Systems	ssc04670	Leukocyte transendothelial migration	0.037
	Environmental Information Processing	ssc04390	Hippo signaling pathway	0.05
	Cellular Processes	ssc04530	Tight junction	0.053
	Environmental Information Processing	ssc04015	Rap1 signaling pathway	0.068
	Environmental Information Processing	ssc04024	cAMP signaling pathway	0.072
	Environmental Information Processing	ssc04014	Ras signaling pathway	0.075
Asian-Pacific pigs	Organismal Systems	ssc04976	Bile secretion	<0.001
	Metabolism	ssc00053	Ascorbate and aldarate metabolism	<0.001
	Metabolism	ssc00040	Pentose and glucuronate interconversions	<0.001
	Metabolism	ssc00860	Porphyrin metabolism	0.001
	Metabolism	ssc00982	Drug metabolism - cytochrome P450	0.001
	Metabolism	ssc00980	Metabolism of xenobiotics by cytochrome P450	0.002
	Human Diseases	ssc05204	Chemical carcinogenesis - DNA adducts	0.002
	Metabolism	ssc00140	Steroid hormone biosynthesis	0.002
	Metabolism	ssc00983	Drug metabolism - other enzymes	0.002
	Metabolism	ssc00830	Retinol metabolism	0.002
	Metabolism	ssc01240	Biosynthesis of cofactors	0.01
	Human Diseases	ssc05207	Chemical carcinogenesis - receptor activation	0.019
	Organismal Systems	ssc04964	Proximal tubule bicarbonate reclamation	0.024
	Environmental Information Processing	ssc04392	Hippo signaling pathway - multiple species	0.03
OBP1	Human Diseases	ssc05213	Endometrial cancer	0.001
	Organismal Systems	ssc04211	Longevity regulating pathway	0.003

A total of 16, 24, 4, and 8 GO terms were significantly enriched for European and North American Duroc, Australian Duroc, OBP1, and OBP2 pigs, respectively (Table 5). No GO terms were enriched for Asian-Pacific pigs.

**Table 5:** Significantly enriched (*P* < 0.05) gene ontology (GO) terms identified for the breed groups

**Group**	**ONTOLOGY**	**ID**	**Description**	***P*-value**
North American / European Duroc	BP	GO:0051321	meiotic cell cycle	<0.001
	BP	GO:1902532	negative regulation of intracellular signal transduction	<0.001
	BP	GO:0051445	regulation of meiotic cell cycle	<0.001
	BP	GO:0051726	regulation of cell cycle	<0.001
	BP	GO:0035329	hippo signaling	<0.001
	BP	GO:2000241	regulation of reproductive process	0.001
	BP	GO:1903046	meiotic cell cycle process	0.001
	BP	GO:0051446	positive regulation of meiotic cell cycle	0.001
	BP	GO:0007260	tyrosine phosphorylation of STAT protein	0.001
	BP	GO:0040020	regulation of meiotic nuclear division	0.001
	BP	GO:0042509	regulation of tyrosine phosphorylation of STAT protein	0.001
	BP	GO:0009968	negative regulation of signal transduction	0.001
	BP	GO:0014013	regulation of gliogenesis	0.001
	BP	GO:0010564	regulation of cell cycle process	0.001
	BP	GO:0010648	negative regulation of cell communication	0.001
	BP	GO:0023057	negative regulation of signaling	0.001
Australian Duroc	BP	GO:0032735	positive regulation of interleukin-12 production	0.003
	BP	GO:0050918	positive chemotaxis	0.003
	BP	GO:0032615	interleukin-12 production	0.004
	BP	GO:0032655	regulation of interleukin-12 production	0.004
	BP	GO:0032732	positive regulation of interleukin-1 production	0.005
	BP	GO:0032612	interleukin-1 production	0.008
	BP	GO:0032652	regulation of interleukin-1 production	0.008
	BP	GO:0032755	positive regulation of interleukin-6 production	0.009
	BP	GO:0030301	cholesterol transport	0.009
	BP	GO:0015918	sterol transport	0.010
	BP	GO:0050729	positive regulation of inflammatory response	0.011
	BP	GO:0050921	positive regulation of chemotaxis	0.011
	BP	GO:0032635	interleukin-6 production	0.014
	BP	GO:0032675	regulation of interleukin-6 production	0.014
	BP	GO:0051924	regulation of calcium ion transport	0.015
	BP	GO:0050920	regulation of chemotaxis	0.017
	MF	GO:0120015	sterol transfer activity	0.003
	MF	GO:0120020	cholesterol transfer activity	0.003
	MF	GO:0015485	cholesterol binding	0.006
	MF	GO:0120013	lipid transfer activity	0.007
	MF	GO:0032934	sterol binding	0.009
	MF	GO:0043178	alcohol binding	0.009
	MF	GO:0005496	steroid binding	0.014
	MF	GO:0005319	lipid transporter activity	0.016
OBP1	MF	GO:0004659	prenyltransferase activity	0.008
	MF	GO:0008320	protein transmembrane transporter activity	0.008
	MF	GO:0140318	protein transporter activity	0.009
	MF	GO:0022884	macromolecule transmembrane transporter activity	0.010
OBP2	MF	GO:0003682	chromatin binding	0.007
	MF	GO:0016702	oxidoreductase activity, acting on single donors with incorporation of molecular oxygen, incorporation of two atoms of oxygen	0.008
	MF	GO:0141052	histone H3 demethylase activity	0.008
	MF	GO:0016701	oxidoreductase activity, acting on single donors with incorporation of molecular oxygen	0.009
	MF	GO:0032452	histone demethylase activity	0.009
	MF	GO:0140457	protein demethylase activity	0.009
	MF	GO:0032451	demethylase activity	0.012
	MF	GO:0017069	snRNA binding	0.012

### FST results

To compare the differences between the Duroc (a terminal sire breed) and Yorkshire pigs (a selected maternal breed), we performed an F_ST_ analysis between these two breeds. For the different Durocs-Yorkshire pairs, genomic regions with the top 1% F_ST_ values were determined as outliers, i.e., the most relevant regions. Like the ROH islands analysis, we also identified shared F_ST_ genomic regions between the European and North American Duroc, Australian Duroc, Asian-Pacific, OBP1, and OBP2 subgroups. Based on the outliers, a total of 13,596 candidate genes were found for the European and North American Duroc– Yorkshire pair of breeds and 17,612 genes for the Australian Duroc—Yorkshire pair, respectively (see Additional file 5, Tables S3 and see Additional file 6, Table S4). Eleven and 36 KEGG pathways were significantly enriched among the identified candidate genes for the European and North American Durocs—Yorkshire pair and the Australian Durocs—Yorkshire pair,, respectively (Table 6). Enriched KEGG pathways for the two pairs were mainly related to immune system functions, disease susceptibility, signal transduction, and metabolism. For the European and North American Durocs—Yorkshire pair, the most significant KEGG pathway was related to nitrogen metabolism. For the Australian Durocs—Yorkshire pair, six disease related pathways (especially cardiovascular disease) were enriched (Table 6). Also, many fatty acid related pathways were enriched, such as cholesterol metabolism, bile secretion, and fatty acid elongation. Five and 18 GO terms were significantly enriched for the European and North American Durocs—Yorkshire and Australian Durocs—Yorkshire pairs, respectively (Table 7). Most of these GO terms were related to cytokine activity and signaling receptor activator activity.

**Table 6:** Significantly enriched (*P* < 0.05) Kyoto Encyclopedia of Genes and Genomes (KEGG) pathways among genes in shared runs of homozygosity for the European and North American Duroc – Yorkshire pair of breeds and the Australian Duroc – Yorkshire pair of breeds

Group	Category	Subcategory	ID	Description	q-value
North American / European Duroc	Metabolism	Energy metabolism	ssc00910	Nitrogen metabolism	0.023
	Environmental Information Processing	Signal transduction	ssc04350	TGF-beta signaling pathway	0.023
			ssc04148	Efferocytosis	0.029
	Organismal Systems	Immune system	ssc04622	RIG-I-like receptor signaling pathway	0.029
	Environmental Information Processing	Signal transduction	ssc04371	Apelin signaling pathway	0.03
	Human Diseases	Infectious disease: viral	ssc05167	Kaposi sarcoma-associated herpesvirus infection	0.032
	Organismal Systems	Digestive system	ssc04979	Cholesterol metabolism	0.032
	Human Diseases	Infectious disease: viral	ssc05163	Human cytomegalovirus infection	0.032
	Environmental Information Processing	Signal transduction	ssc04022	cGMP-PKG signaling pathway	0.04
	Human Diseases	Infectious disease: viral	ssc05165	Human papillomavirus infection	0.04
			ssc04820	Cytoskeleton in muscle cells	0.04
	Metabolism	Metabolism of other amino acids	ssc00430	Taurine and hypotaurine metabolism	0.04
	Organismal Systems	Endocrine system	ssc04925	Aldosterone synthesis and secretion	0.04
Australian Duroc	Human Diseases	Cardiovascular disease	ssc05412	Arrhythmogenic right ventricular cardiomyopathy	0.001
	Metabolism	Metabolism of other amino acids	ssc00430	Taurine and hypotaurine metabolism	0.001
	Organismal Systems	Digestive system	ssc04976	Bile secretion	0.001
	Metabolism	Xenobiotics biodegradation and metabolism	ssc00982	Drug metabolism—cytochrome P450	0.001
	Human Diseases	Cardiovascular disease	ssc05410	Hypertrophic cardiomyopathy	0.001
			ssc04820	Cytoskeleton in muscle cells	0.002
	Human Diseases	Cardiovascular disease	ssc05414	Dilated cardiomyopathy	0.003
	Environmental Information Processing	Signaling molecules and interaction	ssc04061	Viral protein interaction with cytokine and cytokine receptor	0.003
	Genetic Information Processing	Folding, sorting and degradation	ssc04141	Protein processing in endoplasmic reticulum	0.004
	Environmental Information Processing	Signal transduction	ssc04350	TGF-beta signaling pathway	0.004
	Environmental Information Processing	Signaling molecules and interaction	ssc04512	ECM-receptor interaction	0.008
	Organismal Systems	Development and regeneration	ssc04360	Axon guidance	0.008
	Organismal Systems	Circulatory system	ssc04261	Adrenergic signaling in cardiomyocytes	0.011
	Organismal Systems	Immune system	ssc04062	Chemokine signaling pathway	0.012
	Metabolism	Metabolism of cofactors and vitamins	ssc00830	Retinol metabolism	0.014
	Organismal Systems	Circulatory system	ssc04260	Cardiac muscle contraction	0.017
	-		ssc04148	Efferocytosis	0.024
	Metabolism	Lipid metabolism	ssc00062	Fatty acid elongation	0.024
	Metabolism	Lipid metabolism	ssc00140	Steroid hormone biosynthesis	0.024
	Environmental Information Processing	Signal transduction	ssc04151	PI3K-Akt signaling pathway	0.029
	Environmental Information Processing	Signal transduction	ssc04371	Apelin signaling pathway	0.030
	Organismal Systems	Endocrine system	ssc04915	Estrogen signaling pathway	0.030
	Metabolism	Xenobiotics biodegradation and metabolism	ssc00983	Drug metabolism—other enzymes	0.030
	Environmental Information Processing	Signal transduction	ssc04390	Hippo signaling pathway	0.030
	Human Diseases	Infectious disease: bacterial	ssc05150	Staphylococcus aureus infection	0.031
	Human Diseases	Infectious disease: viral	ssc05167	Kaposi sarcoma-associated herpesvirus infection	0.037
	Metabolism	Carbohydrate metabolism	ssc00562	Inositol phosphate metabolism	0.038
	Environmental Information Processing	Signaling molecules and interaction	ssc04060	Cytokine-cytokine receptor interaction	0.038
	Metabolism	Metabolism of cofactors and vitamins	ssc00770	Pantothenate and CoA biosynthesis	0.039
	Organismal Systems	Digestive system	ssc04979	Cholesterol metabolism	0.040
	Human Diseases	Infectious disease: viral	ssc05165	Human papillomavirus infection	0.040
	Metabolism	Amino acid metabolism	ssc00280	Valine, leucine and isoleucine degradation	0.040
	Metabolism	Carbohydrate metabolism	ssc00053	Ascorbate and aldarate metabolism	0.040
	Human Diseases	Drug resistance: antineoplastic	ssc01521	EGFR tyrosine kinase inhibitor resistance	0.042
	Organismal Systems	Immune system	ssc04622	RIG-I-like receptor signaling pathway	0.042
	Environmental Information Processing	Signal transduction	ssc04630	JAK-STAT signaling pathway	0.045

**Table 7:** Significantly enriched (*P* < 0.05) gene ontology (GO) terms among genes in shared runs of homozygosity for the European and North American Duroc-Yorkshire pair of breeds and the Australian Duroc – Yorkshire pair of breeds

Group	ONTOLOGY	ID	Description	q-value
North American / European Duroc	MF	GO:0005125	Cytokine activity	2.15E-05
	MF	GO:0030546	Signaling receptor activator activity	0.001
	MF	GO:0048018	Receptor ligand activity	0.001
	MF	GO:0030545	Signaling receptor regulator activity	0.002
	MF	GO:0005132	Type I interferon receptor binding	0.008
Australian Duroc	BP	GO:0042330	Taxis	0.017
	BP	GO:0006935	Chemotaxis	0.017
	BP	GO:0030030	Cell projection organization	0.017
	BP	GO:0120036	Plasma membrane bounded cell projection organization	0.017
	BP	GO:0002323	Natural killer cell activation involved in immune response	0.023
	BP	GO:0051093	Negative regulation of developmental process	0.031
	BP	GO:0002286	T cell activation involved in immune response	0.031
	BP	GO:0051241	Negative regulation of multicellular organismal process	0.033
	BP	GO:0040011	Locomotion	0.034
	BP	GO:0060322	Head development	0.042
	BP	GO:0150063	Visual system development	0.042
	MF	GO:0005125	Cytokine activity	0.002
	MF	GO:0008289	Lipid binding	0.012
	MF	GO:0042379	Chemokine receptor binding	0.012
	MF	GO:0005132	Type I interferon receptor binding	0.012
	MF	GO:0005126	Cytokine receptor binding	0.012
	MF	GO:0030546	Signaling receptor activator activity	0.022
	MF	GO:0048018	Receptor ligand activity	0.022

## Discussion

The exact origin of the Duroc breed is still unclear. Over generations, Durocs have been exported and imported across countries, leading to variations in their genetic background due to differences in founder populations, selection strategies, and (cross)breeding programs. This is the first genetic diversity and population structure study focusing on a broad assessment of Duroc pigs, representing various North American, European, and Australian corporate breeding programs and traditional purebred breeding entities. As with other species, selective breeding to improve relevant traits has resulted in reduced genetic diversity [35, 36]. Studies on genetic diversity and population structure are essential to design long-term sustainable breeding programs. Several additional commercial pig breeds were also included in this study to better contextualize the Duroc genetic diversity and population structure, including Berkshire, Chester White, Fengjing, Meishan, and Large Black, originating from Asia, Europe, and the Pacific islands.

The greater than expected Ho for Fengjing pigs and the greater than expected He in Hawaiian pigs indicate that there is sufficient genetic variation in Fengjing pigs and greater inbreeding in Hawaiian pigs [37, 38]. The relatively low H_O_ and H_E_ estimates for Asian-Pacific pigs (Meishan, Fengjing, and Hawaiian pigs) were not unexpected. Previous study has indicated that Chinese domestic pigs often show higher He and genetic diversity than European pigs [39]. However, low values have also been reported in previous studies. For instance, Faria et al. [38] reported that Meishan pigs had the lowest He and Ho and other Asian pigs (Fengjing, Minzhu, Hawaii island) had relatively low He (0.339 to 0.353) and Ho (0.322 to 0.374) [38]. Ai et al. [40] and Wang et al. [41] both reported that European and composite breeds had greater H_O_ than most Chinese indigenous breeds. These results may be attributed to ascertainment bias in the genotyping arrays, as the most commonly used SNP panels were designed based on European and North American pig breeds [42]. Furthermore, the small sample sizes used for the studies and the sampling strategies may have under-represented the true genetic variability of Chinese indigenous pigs.

The PCA (Fig. 2) and phylogenetic tree (Fig. 3) analyses provided a high-resolution view of the Duroc breed’s complex history and revealed five distinct subgroups. These results offer strong genomic support for the foundation of the Australian Duroc population from North American Durocs, as these groups clustered closely, reflecting historical germplasm exchanges. Beyond this recent history, our data also revealed a deeper ancestral connection to the OBP1 cluster (comprising Hereford and Tamworth), consistent with the historic use of Duroc sires in development of the Hereford breed. A clear example of these connections is the specific genetic similarity observed among the North American White Duroc (Duroc9), the Australian Duroc, and the OBP1 cluster. The close relationship between the White Duroc and Australian Duroc may be due to the fact that both lines stem from a common North American gene pool from the mid-to-late twentieth century, with their divergence resulting from direct importation versus selective outcrossing. There may also have been introgression of maternal lines (white breeds) into the Australian Durocs over time, which could explain their similarity with the Duroc9 population. The observed similarity with the OBP1 cluster can be explained by its constituent breeds, with the link to Hereford being attributable to its known foundational Duroc ancestry [43], while the connection to Tamworth, for which there is no documented Duroc origin, may reflect shared North American ancestry or unrecorded historic gene flow. By contrast, Asian-Pacific pigs were more distinct, indicating their potentially independent domestication paths and considerable genetic divergence from both the Duroc populations and other commercial breeds.

In phylogenetic tree results, the distinct separation of White Duroc (Duroc9) reflects its composite origin based on Durocs and white pig breeds (e.g., Yorkshire or Landrace), both commonly used in commercial breeding for their desirable traits. The subdivision of Duroc7 suggests that pigs from Duroc7 may be a recent combination of different sub-lines, while the split in Duroc2 may reflect different breeding goals such as their use in the show ring segment of the industry or productivity improvement. There were also significant importations and extensive exchanges of genetic material between North America and Europe (especially Denmark), which is known to have happened with Duroc3 and a proportion of Duroc7, which may explain their closer genetic relationship. The cluster of Duroc4, Duroc5, and Duroc6 was expected, as these three populations are from Canada and may share founder animals.

The admixture analysis showed different ancestry among the Duroc populations. The distinct pattern of Duroc7 and Duroc3 at K = 2 indicates that these two subpopulations may have differed from others due to their selection strategies and potential migration events. The European and North American and Australian Durocs split at K = 3 (except for Duroc3, Duroc7, and Duroc9) implying breeding and selection strategy differences over time. The similarity among Duroc6 through Duroc13 at K = 4 indicates that these corporate lines have not become substantially differentiated over time. Interestingly, these populations are all similar to the Duroc3 group, which includes boars from the NAGP, suggesting the collection has broadly captured the Duroc’s genetic diversity.

More K values were used to test the ancestry among Durocs and other breeds. At lower K values (e.g., K = 2 and K = 4), European and North American Durocs, Australian Durocs, OBP1, and OBP2 exhibited strong similarity, showing that they share a common ancestor with few differences having emerged over time. Australian Durocs and Duroc9 (White Duroc) were more similar compared to the rest of the European and North American Durocs. This could reflect shared ancestry or potential introgression events. Additionally, Asian-Pacific pigs have a more distinct genetic background, influenced by both Chinese indigenous pigs and European pigs. The distinct separation of Duroc subpopulations highlights the need for an industry-driven strategy to conserve the breed's genetic variability. While genetic drift within populations can reduce variation, combining these populations offers a valuable opportunity to restore and enhance genetic diversity in the entire Duroc breed.

Estimates of average ${r}^{2}$ (0.41) for markers with pairwise distance below 100 kb were similar to previous studies (${r}^{2}$ = 0.42) in Durocs [44]. However, differences exist between Duroc subpopulations. Compared to American Durocs, Canadian Durocs (Duroc4, Duroc5, and Duroc6) had relatively higher LD levels, which was also observed by Wang et al. [8]. Lower LD levels were observed in Australian Durocs and Asian-Pacific pigs, such as Hawaiian, Minzhu, Meishan, and Fengjing, implying that they did not lose as much genetic diversity over time as compared to European and North American Durocs. However, this low level of LD may require the use of higher-density SNP panels and larger reference populations for genomic selection and genome-wide association study (GWAS) purposes. In addition, LD decayed faster with distance in the OBP than in the Duroc populations. The different marker distances to reach background LD levels in Durocs (500 kb) and OBPs (300 kb) suggest that Durocs may have experienced more inbreeding over time. This observation aligns with the development of the Duroc breed, which has been intensively used as a terminal sire breed in crossbreeding programs.

D'Augustin et al. [45] used pedigree information of Durocs from multiple herds that were part of the across-herd genetic evaluations of the National Pig Improvement Program in Australia, and estimated Ne to range from 42 to 61 over time, similar to our results. Welsh et al. [46] estimated Ne using pedigree information for Berkshire (Ne = 77.3), Duroc (Ne = 113.1), Hampshire (Ne = 109.2), Landrace (Ne = 74.2), and Yorkshire (Ne = 112.9), which were more similar to our Ne result for the whole Duroc population (Ne = 172). The Ne for the entire Duroc population was greater than that of each Duroc subpopulation, which was as expected as the subpopulations may have diverged over time due to selection, migration (e.g., introgression of other breeds in the subpopulations), and random drift. Faria et al. [38] also observed similar Ne of around 100 for different pig breeds, such as Berkshire, Chester White, and Duroc,. The estimated Ne value at 25 generations ago observed in the Złotnicka White (~ 65) and Złotnicka Spotted (~ 50) pig breeds is more similar to the values observed in our study [47]. Estimates of Ne are largely affected by the methods used, even for the same population. For example, Ne estimates for U.S. Polypay sheep (i.e., single population) ranged from 41 based on the increase in inbreeding by complete generation to 249 based on the increase in inbreeding by maximum generation [48]. Our estimates of Ne were not high for any of the (sub)populations, indicating that caution should be taken to avoid inbreeding and enable long-term genetic progress in pig populations. There may be value in exchanging sires across corporate lines to increase genetic variability within subpopulations and avoid inbreeding.

The low Ne is probably driven by selection and the number of founders used to establish closed populations (e.g., corporate lines). This then leads to increased LD and subsequent fixation of some LD blocks [49]. Khathar et al. [50] mentioned that a sample size of 75 individuals is enough for LD estimation based on the ${r}^{2}$ metric. The small sample size used for the analyses in some of our populations may have led to overestimated LD and underestimated Ne. The number of SNPs after QC can also affect the accuracy of the LD and Ne estimates. In addition, for the corporate lines animals were sampled from the last two generations, while there could have been greater variability in the other populations.

Assessing the loss of genetic variability over time is crucial for maintaining effective breeding programs. ${F}_{HOM}$ was negative for most populations, which may have been due to the small sample sizes used for the analyses, the reduced number of SNPs after QC, and potential ascertainment bias of SNP panels. Random sampling errors may have also contributed to the negative values observed. Mulim et al. [51] observed similar correlation patterns among inbreeding metrics in cattle populations, i.e. high correlations between ${F}_{HOM1}$ and ${F}_{UNI}$, ${F}_{HOM1}$ and ${F}_{HOM2}$, and ${F}_{GRM}$ and ${F}_{UNI}$. The ${F}_{ROH}$ values were the highest among all the inbreeding coefficients and this was also observed previously, including for Duroc [8, 52], Berkshire, Landrace, and Piétrain [53]. ${F}_{ROH(8-16Mb)}$ and ${F}_{ROH(>16Mb)}$ both made a major contribution to ${F}_{ROH}$ of Duroc populations, revealing the more recent inbreeding and increase in homozygosity, which may be related to the implementation of genomic selection in the past two decades. However, it is worth noting that genomic information can be used for more accurate mating designs and, therefore, minimizing the rate of inbreeding. Correlations between ${F}_{ROH}$ and ${F}_{ROH(<2Mb)}$ were low to moderate in Australian Durocs (0.41), OBP1 (0.18), OBP2 (0.46), and Asian-Pacific populations (0.49). ${F}_{ROH}$ can uncover the approximate age of inbreeding based on the length of ROH and have the advantage of not being affected by estimates of allele frequencies, unlike other measures (${F}_{HOM1}$, ${F}_{UNI}$, ${F}_{HOM2}$) [54]. As a breed with intense selection over many generations and controlled matings, the relatively low inbreeding levels of Durocs (average ${F}_{ROH}$ for all Duroc subpopulations = 0.198) were as expected. However, differences in ${F}_{ROH}$ values still exist among Duroc populations, with many (i.e., Duroc7, Duroc8, and Duroc9) showing relatively low genomic inbreeding levels, possibly due to differences in breeding program designs. ROH detection can also be affected by the marker density used [54], which may partially explain the lowest ${F}_{ROH}$ value in the Duroc7 population, which had the smallest number of SNPs after QC. Shorter ROHs significantly contributed to ${F}_{ROH}$ of all the studied populations, especially of the non-Duroc populations. Future studies on these populations with larger density SNP panels or whole-genome sequence data would be valuable to validate these results.

The ROH number, length, and distribution can provide information on the levels of population size reduction, inbreeding events, demography history, and natural or artificial selection [55]. Significant variation in recent inbreeding was observed within the Duroc breed. In particular the Duroc2, Duroc3, Duroc4, and Duroc6 populations stood out, each showing a very high number and proportion of individuals with long ROHs (> 8 Mb). In addition, maximum ROH lengths exceeded 100 Mb in the Duroc2, Duroc3, and Duroc4 populations, strongly indicating that these subpopulations are undergoing more intensive selection and mating of closely related individuals compared to most OBP subpopulations. When averaged across all subpopulations, the mean proportion of long ROHs (> 8 Mb) was similar in Duroc and OBP populations, indicating comparable levels of recent inbreeding but likely forvery different reasons. In Durocs, high levels of long ROHs are the direct results of deliberate, intensive selection and line-breeding, aimed at maximizing genetic gain. In contrast, for OBPs, this pattern is more likely an unavoidable outcome of their demographic reality, including critically small population sizes and closed-herd conservation strategies that lead to mating among related individuals. This high level of recent inbreeding poses a significant threat to the long-term viability of these breeds, elevating the risk of inbreeding depression and genetic diversity reduction.

Among all subpopulations, Duroc3 had the greatest total number of ROHs (n = 15,648), far exceeding that of other Duroc lines and OBP breeds. In contrast, breeds like Yorkshire also had high ROH counts (11,299 segments), but all OBPs were dominated by short segments (< 2 Mb) compared to Durocs, pointing to sustained genetic drift in a large but historically bottlenecked population rather than to very recent inbreeding. Conversely, Duroc subpopulations had a higher proportion of individuals carrying ROHs between 2 to 4 Mb, potentially reflecting targeted line-breeding without reaching critical inbreeding thresholds.

While Ceballos et al. [55] showed that populations with smaller Ne tend to have more ROH, our results revealed notable exceptions. For example, the Duroc4 line (Ne = 33) showed the highest ROH count, whereas Duroc12 (Ne = 28) displayed the lowest ROH frequency. Although sample size and sampling strategy (for genotyping) can influence ROH detection, these findings clearly suggest that additional factors beyond long-term Ne significantly influence ROH patterns in commercial pig lines.

While the number of very long ROHs (> 16 Mb) in Duroc3 (n = 87) was notably lower than in some subpopulations like Duroc4 (n = 534) or Meishan (n = 295), its exceptionally high counts of shorter and medium-length ROH segments were remarkable. Most individuals in the Duroc3 subpopulation exhibited a total ROH length exceeding 800 Mb and a total ROH count above 120, as shown in Fig. 8b. This is supported by the highest ${F}_{ROH}$ value in the Duroc3 subpopulation. These findings point to a need for more effective inbreeding management strategies within the Duroc3 subpopulation. This also indicates an ancient reduction in genetic diversity and loss of homozygosity in the Duroc subpopulations. Pemberton et al. [56] reported that, recent strong directional selection may have a greater impact on long ROH than on medium and small ROH.

The origins of ROH islands are still debated, but they seem to result from extended haplotypes segregating at high frequencies within these regions in the population [55]. A ROH island typically forms when an allele increases in frequency due to selection for certain traits and the genomic regions that overlap with these ROH island regions tend to have low recombination rates [55]. In European and North American Durocs, *UQCR10* encodes a component of mitochondrial complex III, a critical element of the inner mitochondrial membrane’s electron transport chain. This gene has been previously reported to be related to intramuscular fat accumulation [57, 58] and feed efficiency [59] in pigs. The *VDAC1* gene mediates mitochondrial ATP/ADP exchange, influencing sperm motility, survival rate, fertilization, and embryo development [60–62]. Its fixation in a homozygous region likely reflects selection for improved semen quality. This underscores fertility as a critical economic trait targeted in modern boar breeding [9]. The *ADAMTSL3* gene was identified in ROH islands of both European and North American and Australian Durocs and has been identified as a strong selection signature in swine [63, 64]. It has also been associated with feed intake [65] in pigs and is considered an important candidate gene for body length [64, 66].

Twenty-three genes overlapped with ROH islands on SSC8 for Asian-Pacific pigs. These genes are associated with important functions such as lactation (*CSN2*, *CSN3* [67, 68]) and growth (*MOB1B* [69]). We also identified genes indicative of adaptive selection, such as the Vitamin D binding protein gene *GC* [70], which may aid in calcium utilization with high-fiber diets [71], and *TMPRSS11D*, which has been linked to susceptibility to influenza [72]. These genes suggest strong adaptive selection for physiological robustness and disease resistance in their local environments.

Many genes on different chromosomes (SSC1, SSC2, and SSC14) were identified for ROH islands that were shared by OBP1 pigs, and many of these were previously related to growth (*ARMC2*, *AFG1L*, *NR2E1*) [73–75], longevity (*FOXO3*) [76], and cellular stress-response (*SESN1*) [77]. In OBP2 pigs, *JMJD1C* has been shown to regulate de novo lipogenesis [83] and is essential for the long-term maintenance of germ cells in mice [78], while *SNRPA1* has been linked to overall fitness and reproductive traits [79].

These KEGG pathways that were enriched within the ROH islands of the Australian Duroc population show that selection in this population has focused on growth and meat quality. Hippo, Ras, and Rap1 signaling pathways can regulate muscle cell proliferation [80], cell differentiation [81], and apoptosis [82, 83], leading to enhanced muscle growth. Ras and Rap1 signaling pathways have been found to be enriched in genomic regions associated with feed efficiency in Durocs before [84]. Previous study has shown that ribosome biogenesis appears to be a key process for increasing protein synthesis and cell growth [85].

The KEGG pathways that were enriched in ROH islands of Asian-Pacific pigs highlight strong environmental adaptation through detoxification and lipid metabolism. Enrichment of cytochrome P450 and xenobiotic-metabolism pathways suggests adaptation to plant-based toxins in their local diets [86]. Independently, steroid-hormone biosynthesis and bile-secretion pathways, both of which are derived from cholesterol, were also enriched, indicating that the entire lipid-processing network has been under selection to support efficient fat digestion and storage [87]. Enrichment of retinol metabolism reinforces this lipid-focused adaptation, since fat-soluble nature of vitamin A ties its uptake and storage directly to lipid pathways [88]. The ascorbate and aldarate metabolism pathway, although not a lipid pathway per se, likely contributes antioxidant protection against oxidative stress caused by high-fat diets [89]. Together, these findings align with observations that native Asian breeds (e.g., Bamei, Meishan) have lower growth rates but higher fat deposition than commercial breeds [90–94], illustrating selection for a robust metabolic system capable of both detoxifying diverse feeds and managing high-fat accumulation.

To compare candidate genes under selection between a maternal line (Yorkshire) and a terminal sire line (Duroc), F_ST_ analysis was used to assess population differentiation. This differentiation could result from a combination of natural and artificial selection on different breeding criteria. Four KEGG pathways were identified in both the North American—Yorkshire pair and the Australian Duroc—Yorkshire pair, including the TGF-beta signaling pathway, efferocytosis, Taurine and Hypotaurine metabolism, and Cytoskeleton in muscle cells. The shared pathways in both these population comparisons emphasize common genetic features related to immune function and muscle maintenance, while the differences highlight distinct genetic influences on each population.

ROH islands for European and North American Durocs were enriched for pathways related to immune responses, viral infections, and metabolic processes, indicating adaptations to different pathogens and dietary factors or resilience to viral infections. In contrast, ROH islands for Australian Durocs were enriched for pathways associated with lipid metabolism, cardiac health, stress responses, and drug metabolism, suggesting genetic predispositions to heart conditions and adaptations to environmental stressors and xenobiotics. The lipid metabolism related pathways could positively affect feed intake and weight gain [95, 96]. While Duroc pigs are well-known for their high growth rate, high glycolytic fiber content, and low intramuscular fat content [97], Yorkshire pigs are recognized for their high number of piglets born alive, high proportion of lean meat and low backfat [98].

These results indicate significant differences in genetic diversity, population structure, and ancestry among Duroc subpopulations and between Duroc and other breeds, which can be attributed to genetic drift, potential migration events, and differential selection strategies. Clear genetic differentiation was found among Duroc subpopulations, reflecting differences breeding strategies between selection programs (e.g., selection objectives, selection intensities, initial size of the founder populations). The separation of Duroc subpopulations supports that there is large genetic variability within the breed across the globe if the subpopulations were combined or if other populations were formed by combining animals from all Duroc subpopulations (i.e., migration across subpopulations). This is supported by the Ne estimate for the entire Duroc population (Ne = 172). It indicates that the closed Duroc subpopulations could exchange genetic material to increase genetic variability within their breeding programs, as there is considerable variation across subpopulations. The Food and Agriculture Organization of the United Nations (FAO) has recommended a goal of maintaining the rate of increase in inbreeding below 1% per generation and an Ne greater than 50 [99]. The high LD levels and small Ne values observed in Durocs and other pig populations suggest the need to monitor rates of inbreeding and develop strategies for maintaining genetic diversity levels over time, including the exchange of genetic material across Duroc subpopulations. The enrichment results for shared ROH islands and F_ST_ from pig subgroups provide new insights into how genetic information has been influenced by selection over generations, identifying many immune response, energy, and metabolic pathways in each subgroup.

## Conclusions

Durocs represent an interesting model of how global breeds may be composed of an array of subpopulations that harbour genetic variation. Over time, corporate lines have, and we assume continue to, become divergent, although similar selection objectives are likely used. The separation of Duroc subpopulations suggests a de-facto industry-based approach for conserving this breed’s genetic resources. While individual populations are subject to genetic drift (due to selection and chance), there is the opportunity to increase genetic variation by combining subpopulations. The relatively low Ne estimates and high LD levels in subpopulations of Durocs suggest that subpopulation management should be improved to mitigate potential inbreeding depression and related problems. The ROH islands in each subgroup contained many genes associated with reproductive traits, muscular development, fat deposition, and adaptation. Genetic differences and selection signatures were found to exist when comparing Duroc to commercially important breeds like Yorkshire and to small and isolated populations found in Asia and the Pacific region. As a result, the Duroc breed appears to be positioned to continue its contribution to global pork production.

## Supplementary Information

Additional file 1 Cross validation error of each K values in the admixture analysis for (a) Duroc pigs and (b) all pigs

Additional file 2 Number of runs of homozygosity in pig populations according to the length (a) in Duroc pigs and (b) other selected pigs

Additional file 3 Effective population size of each pig population from generation 100 to 25 ago

Additional file 4 Identified genes within shared runs of homozygosity islands for each pig group

Additional file 5 Significantly enriched (P < 0.05) genes identified for European and North American Durocs - Yorkshire pair in Fst analysis

Additional file 6 Significantly enriched (P < 0.05) genes identified for Australian Durocs - Yorkshire pair in Fst analysis

## Data Availability

The data and results that support this study's conclusions are available in the paper and its additional files.
