# Supplementary figures and images for: Characterization of genomic diversity and population structure of worldwide Duroc subpopulations and other pig breeds
Source: Genet Sel Evol. 2025 Dec 16;58:1. doi: 10.1186/s12711-025-01017-6 (PMC12781363; doi:10.1186/s12711-025-01017-6)

A

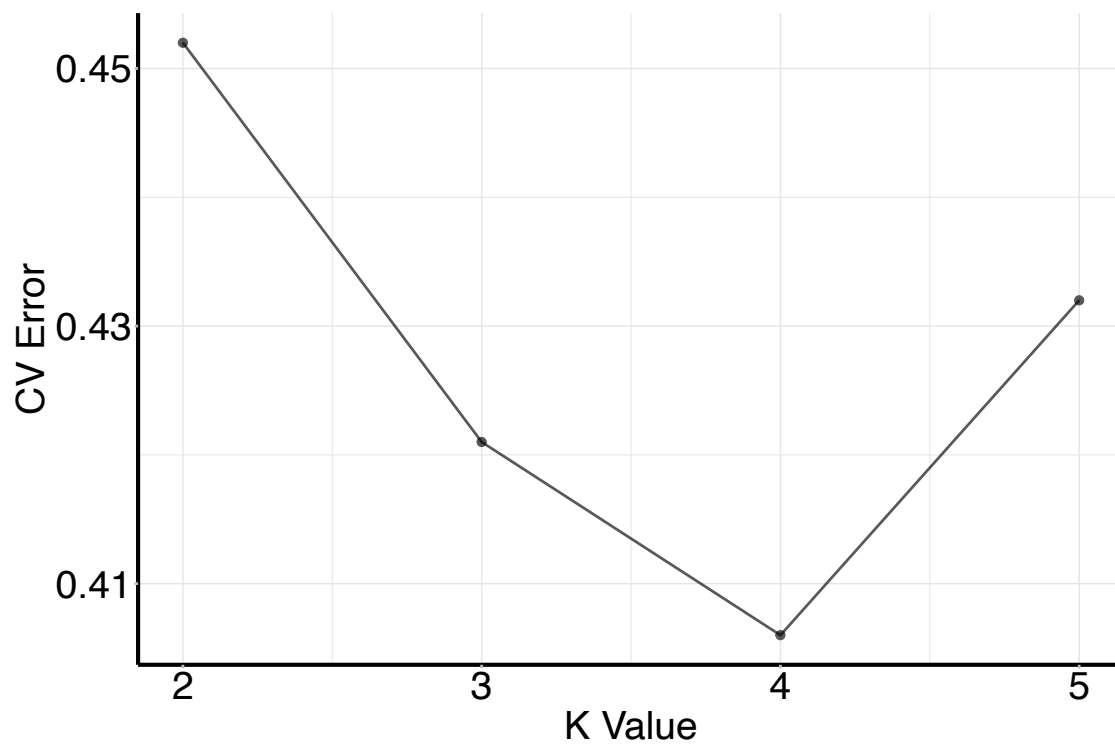

B

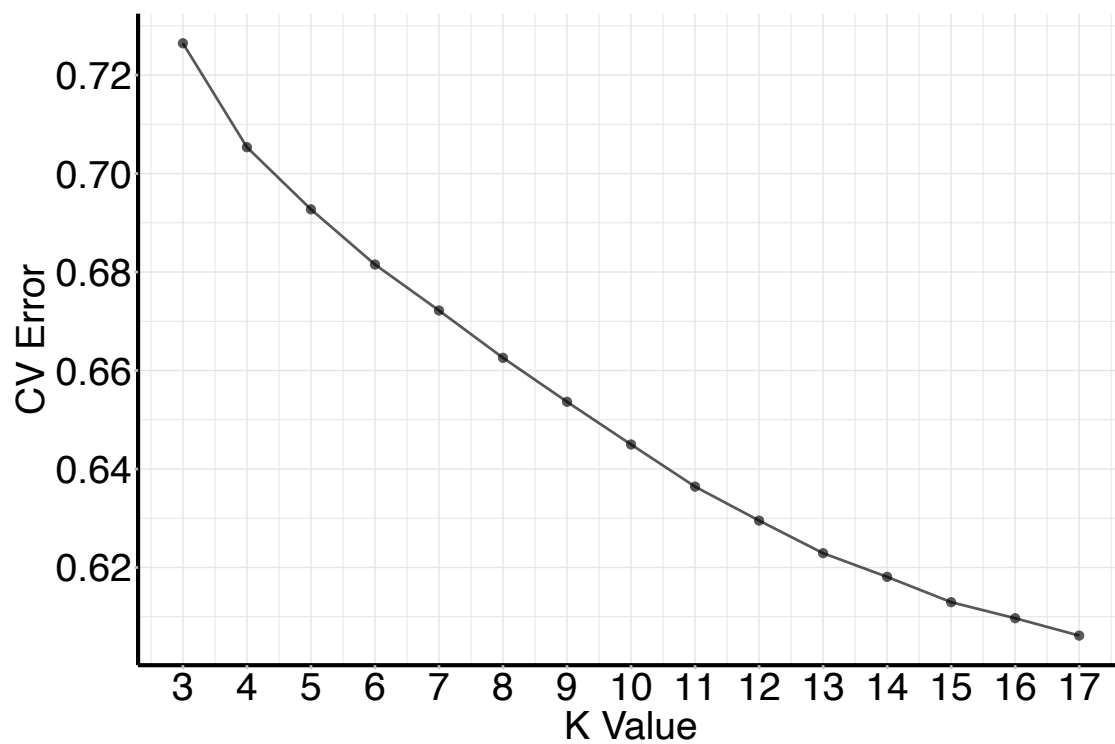

Supplement: Supplementary file 1 — Additional file 1 Cross validation error of each K values in the admixture analysis for (a) Duroc pigs and (b) all pigs [file 12711_2025_1017_MOESM1_ESM.pdf]

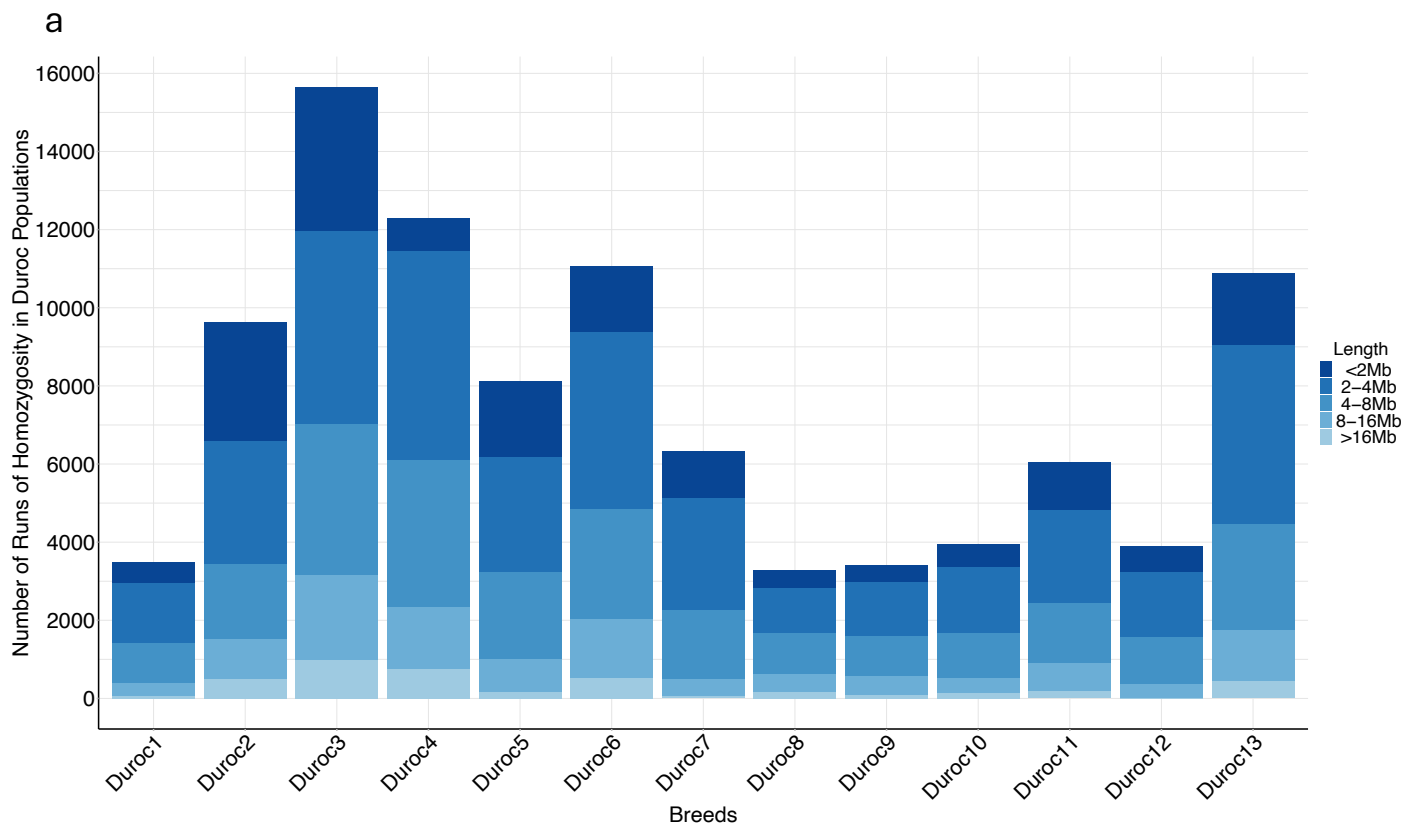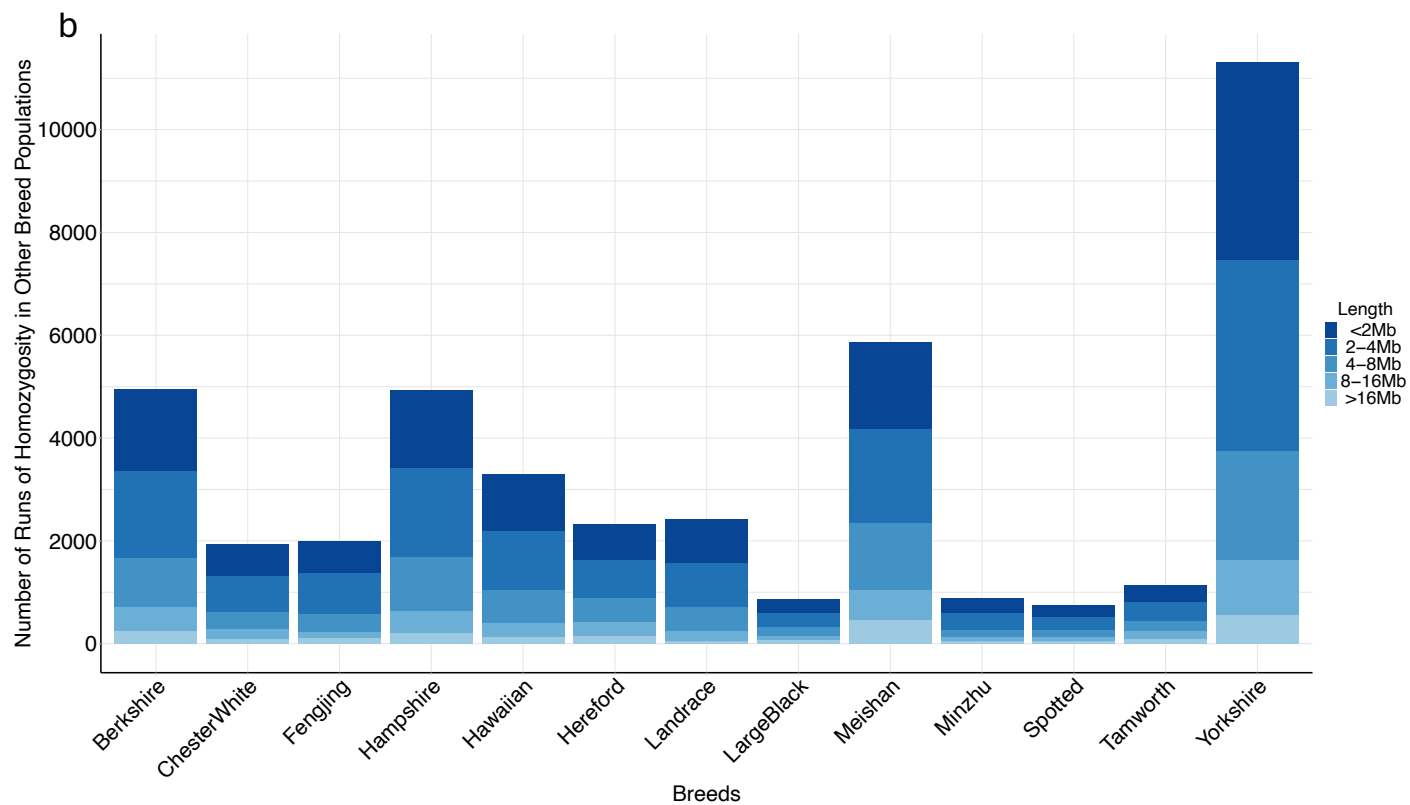

Supplement: Supplementary file 2 — Additional file 2 Number of runs of homozygosity in pig populations according to the length (a) in Duroc pigs and (b) other selected pigs [file 12711_2025_1017_MOESM2_ESM.pdf]
